# Using generalized additive models to decompose time series and waveforms, and dissect heart–lung interaction physiology

**DOI:** 10.1007/s10877-022-00873-7

**Published:** 2022-06-13

**Authors:** Johannes Enevoldsen, Gavin L. Simpson, Simon T. Vistisen

**Affiliations:** 1 Department of Clinical Medicine, Aarhus University, Palle Juul-Jensens Boulevard 82, 8200 Aarhus N, Denmark; 2 Department of Anaesthesiology and Intensive Care, Aarhus University Hospital, Palle Juul-Jensens Boulevard 99, 8200 Aarhus N, Denmark; 3 Department of Animal Science, Aarhus University, Tjele, Denmark

**Keywords:** Hemodynamic monitoring, Central venous pressure, Mechanical ventilation, Signal processing, Statistical modelling

## Abstract

Common physiological time series and waveforms are composed of repeating cardiac and respiratory cycles. Often, the cardiac effect is the primary interest, but for, e.g., fluid responsiveness prediction, the respiratory effect on arterial blood pressure also convey important information. In either case, it is relevant to disentangle the two effects. Generalized additive models (GAMs) allow estimating the effect of predictors as nonlinear, smooth functions. These smooth functions can represent the cardiac and respiratory cycles’ effects on a physiological signal. We demonstrate how GAMs allow a decomposition of physiological signals from mechanically ventilated subjects into separate effects of the cardiac and respiratory cycles. Two examples are presented. The first is a model of the respiratory variation in pulse pressure. The second demonstrates how a central venous pressure waveform can be decomposed into a cardiac effect, a respiratory effect and the interaction between the two cycles. Generalized additive models provide an intuitive and flexible approach to modelling the repeating, smooth, patterns common in medical monitoring data.

**Supplementary Information:**

The online version contains supplementary material available at 10.1007/s10877-022-00873-7.

## Introduction

Medical waveforms of physiological measurements, like electrocardiogram (ECG), invasive arterial blood pressure (ABP), photoplethysmogram (pleth) and central venous pressure (CVP), are ubiquitous in settings with closely monitored patients, notably in intensive care units and operating rooms. While waveforms of these signals are often displayed on a bedside monitor, they are rarely interpreted directly by the clinician (the ECG being a notable exception). Instead, simple summary characteristics, e.g. heart rate, respiratory rate and standard blood pressure features, are automatically calculated by the bedside monitor and presented beside the waveforms.

The main signal in these waveforms comes from the heart. In addition, respiration impacts the waveform, and the cyclic respiratory effect can convey important information about patient physiology. This is especially recognised in fluid responsiveness research where “dynamic” fluid responsiveness indicators such as the pulse pressure variation (PPV) have repeatedly outperformed “static” indicators [1, 2]. However, the details of the cyclic respiratory effects can be difficult to disentangle, illustrated by the ventilation-related limitations to PPV such as tidal volume, respiratory rate and respiratory system compliance [3].

Researchers have developed several, methods for analysing medical waveforms and derived time series: e.g. pulse pressure variation (PPV), cardiac output estimation, hypotension prediction index, etc. While many of these measures are useful and often implemented in commercial monitors, they do not always reflect what the clinician expects them to (e.g. a high PPV from a patient with a subtle arrhythmia). Generally, these complicated algorithms are difficult to understand and typically proprietary. This makes it difficult for the clinician to critically consider the algorithm’s analysis of the waveform.

The task of analysing physiological data both comprehensively and transparently seems a perfect fit for generalized additive models (GAMs). A recent paper by Wyffels et al. demonstrates how GAMs can be used to isolate the respiratory component of PPV in subjects with atrial fibrillation [4]. An elegant solution that may be used to guide fluid therapy in this patient group.

The aim of this paper is to demonstrate how GAMs can be used to decompose waveforms or time series recorded in mechanically ventilated patients into separate, physiologically relevant, components. This allows analysts to focus on each component individually. We give a short introduction to splines and GAMs, and then demonstrate the method using two examples. First, we use a time series of pulse pressure measurements to give a robust estimate of PPV in mechanically ventilated patients with sinus rhythm (a simplified version of the model presented by Wyffels et al. [4]). Second, we decompose the CVP waveform into separate, physiologically relevant, effects. Finally, we summarise and discuss how GAMs might be used in future research and in clinical monitoring.

### What is a GAM?

Generalized additive models are both flexible and interpretable. In the space of statistical models, they reside somewhere between simple but rigid methods like linear regression and flexible but complex methods like neural networks. With GAMs, we can build transparent models, with components that represent known physiology.

Hastie and Tibshirani introduced GAMs in 1986, as extensions of generalized linear models [5]. Instead of fitting straight lines, GAMs can fit any smooth function. In the basic form of a GAM, a smooth function is fitted for each independent variable in the model. These functions are added together to give the model’s prediction of the dependent variable:

$${Y}_{predicted}=\alpha +f\left({X}_{1}\right)+f\left({X}_{2}\right),$$

where $\alpha$ is a constant value and $f$ can be any smooth function (continuous and with no kinks). In this paper, we do not introduce link functions, and we mainly use models with a Gaussian conditional distribution.

#### Cubic splines

**Fig. 1:**
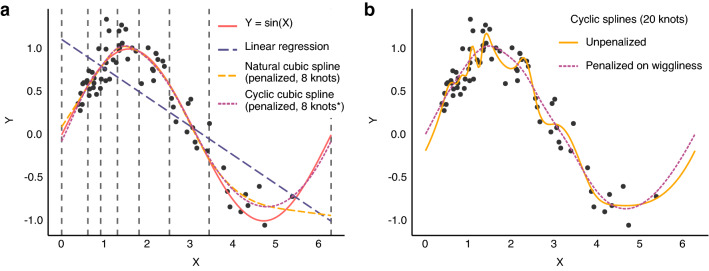
Splines fitted to simulated data (n = 70). The data-generating function is Y = sin(X) with added normally distributed noise. **a** Vertical dashed lines show the position of the 8 knots. *In the cyclic spline there are effectively 7 knots, since the first and last line represent a single knot, joining the ends of the spline. **b** Comparison of a penalised and an unpenalised spline fitted to the same data. The unpenalised spline with 20 knots is clearly too wiggly and overfits the data. Penalising the spline on wiggliness reduces the risk of overfitting, but keeps the model flexible in case the data demand it

Several types of smooth functions can be used to fit data. In this paper, we use one type: the *cubic spline*. A cubic spline is built by combining a number of third-order polynomials. Each polynomial fits its individual section of the data (e.g., a period of time if time is the independent variable) and is constrained to join smoothly to the adjacent polynomial(s). The intersections between adjacent polynomials are called *knots*. Smoothness at the knots is ensured by constraining adjacent polynomials to align at the knots. Specifically, the values of adjacent cubic polynomials’ 0th, 1st and 2nd derivatives must be equal at the knots. The knots can be placed at will, but a common choice is to position knots at the quantiles (including at minimum and maximum) of the independent variable, giving the same number of observations in each segment (see Fig. 1a). Cubic splines are often additionally constrained by fixing the second and third derivative at the outer knots to zero (making them linear outside the outer knots). This is termed a *natural cubic spline* [6].

To reduce the risk of overfitting, splines can be *penalised* according to their wiggliness (by default defined as the integral of the squared 2nd derivative). A penalised spline is fitted to optimise the tradeoff between goodness of fit (e.g. high likelihood) and complexity (measured by the wiggliness of the function) (see Fig. 1b). The relative weight of fit and wiggliness in this tradeoff is controlled with a *smoothing parameter*. This smoothing parameter can be automatically optimised to prevent overfitting (e.g. using a *restricted maximum likelihood* approach [7]) or be chosen manually. A manual smoothing parameter can be useful if there is prior knowledge about the smoothness of one or more splines in the model (e.g. the effect of ventilation is expected to be very smooth).

#### Modelling interaction between variables

Interaction terms can be included in two principal ways. In the simplest case, one term is continuous (${X}_{1}$) and one is categorical (${X}_{2}$). Individual smooth functions are then fit for each category [$f\left({X}_{1}\right)$ for each ${X}_{2}$]. If both terms are continuous, the interaction can be represented as $f\left({X}_{1},{X}_{2}\right)$: a function that takes two values and returns one value. This can be visualised as a smooth plane where each combination of ${X}_{1}$ and ${X}_{2}$ corresponds to an output (the elevation of the plane) (see Fig. 5e.1).

#### Modelling cyclic data

Some variables repeat cyclically without a marked distinction between the end of one cycle and the beginning of the next. An example is compass direction, where 0° ≡ 360°. Likewise, we expect CVP at the end of one respiratory cycle to continue smoothly into the next cycle. We can model the effect of a cyclic variable with a *cyclic cubic spline*. A cyclic cubic spline is a special case of the cubic spline where the first and last knot are treated as one. The beginning and end are effectively adjacent, and the respective splines match up to the 2nd derivative (see Fig. 1a).

## Examples

Examples are analysed using R 4.1.0 [8] with packages: *mgcv* 1.8–36 [7], *gratia* [9] and *tidyverse* [10]. While the paper aims to be language agnostic, sample data and annotated R code are supplied in Online Resource 1 (https://doi.org/10.5281/zenodo.6375221).

### Example data

The data for these demonstrations are recorded during abdominal surgery from three consenting patients on pressure control ventilation (recorded as part of a project registered on ClinicalTrials.gov, NCT04298931 with regional ethical committee approval, case: 1-10-72-245-19). Haemodynamic waveforms (125 Hz) were recorded from a Philips MX550 using Vital Recorder [11] and ventilator data (timestamps for each inspiration start) were recorded from a Dräger Perseus A100 using VSCaputureDrgVent [12].

### Example 1: Pulse pressure

In recent years, more complex waveform analysis is being implemented in the monitors. One example is ventilator-induced pulse pressure variation (PPV): a measure commonly used to predict fluid responsiveness [13]. While it is possible to manually calculate PPV from an arterial pressure waveform, it is neither trivial nor reproducible. Also, manually calculated PPV may differ substantially from the PPV automatically calculated by the monitor. This is due to a sophisticated analysis of the arterial waveform that takes multiple respiratory cycles into account [14, 15]. The PPV calculated automatically by, e.g., Philips monitors is robust to noise and outliers [14], but the steps between the ABP waveform and the automatically calculated PPV are probably unclear to most clinicians.

**Fig. 2:**
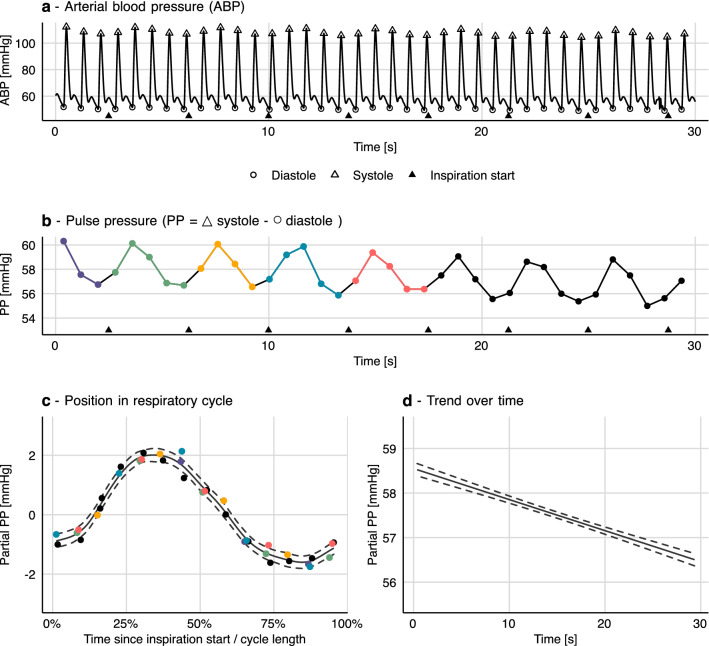
How a generalized additive model (GAM) can be fitted to a series of pulse pressure measurements (derived from the arterial waveform). **a** and **b** For each beat, systolic and diastolic pressure are detected, and pulse pressure (PP) is calculated. A GAM with two smooths **c** and **d** is fitted to the PP time series (**b**). **c** This first smooth represents the variation in PP explained by the beats’ position in the respiratory cycle. Coloured points (beats) correspond between panels **b** and **c**. **d** The second smooth represents the trend in PP over time with the model constant (α) added. The sum of these two smooths (**b** and **c**) gives the model prediction. Residuals of the model (ε) are the vertical distance from the smooth to the points in panel **c** (i.e. the scatters are partial residuals). Dashed curves represent 95% confidence intervals

In the individual, pulse pressure (PP = systolic pressure − diastolic pressure) is highly correlated with stroke volume; and like stroke volume, PP varies between heart beats. The main cause of the short-term variation in PP is respiration, and the effect is especially pronounced during controlled mechanical ventilation. A beat’s position in the respiratory cycle is associated with a specific effect on PP (see Fig. 2c). Around the end of the inspiration, PP is above average; and during expiration, it drops below average (the phase depends on respiratory cycle length).

Variation in pulse pressure (PP) can be understood as the sum of three separate effects. First, the effect of ventilation: with each breath, PP rises and then decreases. This is caused by the breath’s combined effect of both preload and afterload on both ventricles [13]. It is the size of this effect that is related to the response to fluid therapy. Second, PP varies over longer periods, e.g. with changes in vascular tone. Third, there is also a fast, effectively random, variation in PP: e.g. measurement noise and subtle ‘random’ fluctuations in cardiac contractility). This decomposition of PP into three separate effects can be described with the equation:

$$PP=\alpha +f\left({pos}_{ventilationcycle}\right)+f\left(time\right)+\epsilon .$$

$f\left({pos}_{ventilationcycle}\right)$ describes the relationship between a heart beat’s position in the respiratory cycle and the produced PP at that heart beat. $f\left(time\right)$ represents the trend in PP over time, and $\alpha$ is the mean PP over the entire sample. $\epsilon$ represents the remainder: noise, ‘random’ fluctuation, etc.

The individual observations in this analysis are heart beats. For each heart beat, we need to know the time it occurred, its position in the respiratory cycle (time since the start of the latest inspiration/respiratory cycle length) and the pulse pressure of the beat. The timing of each beat was assigned the time of the diastole,^1^ and pulse pressure was calculated as systolic minus diastolic pressure (see Fig. 2a and b). With this data, the model can be fitted as a GAM where $f\left({pos}_{ventilationcycle}\right)$ is a cyclic cubic spline and $f\left(time\right)$ is a natural cubic spline.

After fitting the model, we can inspect the model by plotting the smooth functions over a relevant interval (usually the interval containing the original observations) (see Fig. 2c and d). In our model of pulse pressure, $f\left({pos}_{ventilationcycle}\right)$ represents the variation in pulse pressure with each respiratory cycle. Thus, we can use $f\left({pos}_{ventilationcycle}\right)$ to calculate PPV.

$$PPV=\frac{max\left(f\left({pos}_{ventilationcycle}\right)\right)-min\left(f\left({pos}_{ventilationcycle}\right)\right)}{\alpha },$$

where ${pos}_{ventilationcycle}$ is between 0 and 100%.

Since α is the mean PP, this is equivalent to the classic formula for PPV:

$$PPV=\frac{P{P}_{max}-P{P}_{min}}{\left(P{P}_{max}+P{P}_{min}\right)/2}.$$

Calculation of a confidence interval for PPV is described in Online Resource 1.

**Fig. 3:**
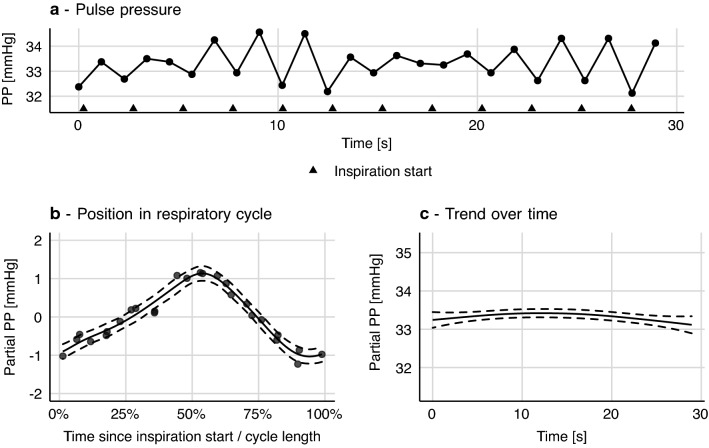
This patient has a heart-rate-to-respiratory rate ratio just beyond 2:1 (52:24). **a** From the pulse pressure (PP) plot, it is difficult to assess pulse pressure variability (PPV), and it seems to be changing. **b** When PP is modelled as a smooth function of each beat’s position in the respiratory cycle, a tight relationship between respiration and PP is revealed. Dashed curves represent 95% confidence intervals

Essentially, a GAM facilitates the “step” from panel b to panel c in Fig. 2, where the highly deterministic effect of heart–lung interactions on pulse pressure is uncovered. Calculating PPV from a GAM model takes every beat in our sample into account. This makes the PPV estimate less sensitive to outliers (min and max being inherently very sensitive to outliers). Also, PPV estimated from individual respiratory cycles will tend to be lower than PPV calculated from a GAM, by a somewhat random amount. Heart beats occur at varying positions in the respiratory cycle; often not at the positions giving both the maximum and minimum pulse pressure. This is especially important in conditions with few beats per ventilation [17] (see Fig. 3). Details about the shape and phase of $f\left({pos}_{ventilationcycle}\right)$ may also contain important information about the heart–lung interaction, though this has not yet been investigated.

### Example 2: Central venous pressure

**Fig. 4:**
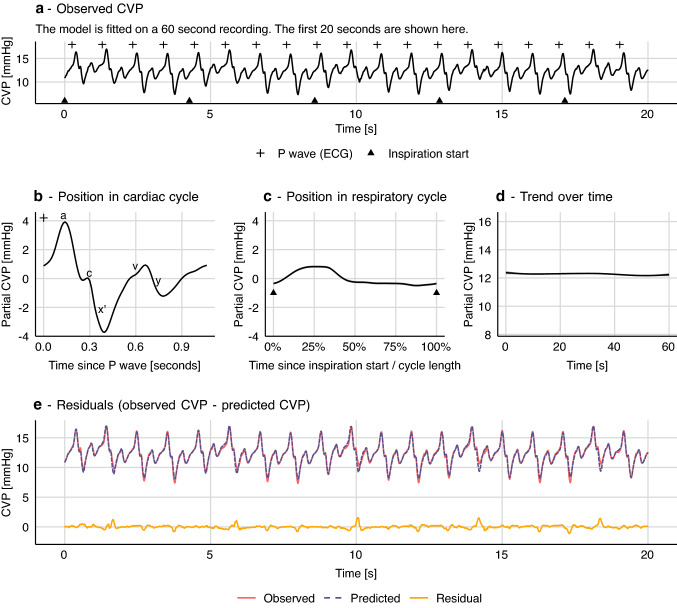
Generalized additive model of central venous pressure (CVP). Variation in CVP is explained by the effects of the cardiac cycle and the respiratory cycle. In this model there is no interaction between the two effects. Grey shades in **b**, **c** and **d** represent 95% confidence intervals (often too narrow to be visible)

Hemodynamic waveforms are affected by both the heart and the lungs. The CVP waveform has a fast period with the length of one cardiac cycle and a slower period with the length of one respiratory cycle. For each cardiac cycle, well-defined features represent atrial contraction (*a*), tricuspid valve closing (*c*), ventricular contraction (*x’*), atrial filling during ventricular systole (*v*) and tricuspid valve opening (*y*) [18, 19] (CVP landmarks are shown in Fig. 4b). If the patient is on a ventilator, the entire CVP waveform will rise with the inspiration and fall with the expiration (see Fig. 4a). A third effect is the interaction between the cardiac cycle and the respiratory cycle. A cardiac cycle during inspiration produces a CVP waveform that is different from what is produced during expiration. Lastly, a number of factors influence CVP and can change over longer periods. These include, but are not limited to: surgical activity, autonomic regulation and medication.

In this example, we model the entire waveform; not just a time series of derived measurements as in the above example with pulse pressure. The unit observations are individual samples of a 125 Hz CVP recording. Each sample has a value (CVP) and a time. Using this sample time, the timing of P waves from the ECG and the timing of each inspiration start, we can compute two additional features: the sample’s position in the cardiac cycle (time since the latest P wave) and its position in the respiratory cycle (similar to example 1). Timing of P waves was calculated by subtracting a constant, manually measured, PR interval from algorithmically determined QRS complex timings. The exact length of the subtracted interval is not very important. It simply ensures that the atrial contraction is placed in the beginning of a cardiac cycle rather than in the end of the previous cycle. We model the effect of the cardiac cycle with a non-cyclic spline, since cardiac cycles vary slightly in length (due to respiratory sinus arrhythmia).

A first approach to modelling CVP from these three features could be a simple extension of the PP model proposed in example 1:

$$CVP=\alpha +f\left({pos}_{cardiac}\right)+f\left({pos}_{ventilation}\right)+f\left(time\right)+\epsilon$$

This is a strictly additive model and therefore assumes no interaction between the effect of ventilation and heart beat on CVP; i.e. this model assumes that every heartbeat produces the same CVP pattern. This pattern is simply raised and lowered with ventilation (see Fig. 4).

This model describes most of the variation in CVP, but the depth of the *x’* descent (corresponding to the ventricular contraction) is systematically off at specific places in the respiratory cycle. Clearly, the pattern in CVP produced by a heart beat depends on its position in the respiratory cycle. To address this, we introduce a smooth interaction term to the model.

$$CVP=\alpha +f\left({pos}_{cardiac}\right)+f\left({pos}_{ventilation}\right)+f\left({pos}_{cardiac},{pos}_{ventilation}\right)+f\left(time\right)+\epsilon .$$

**Fig. 5:**
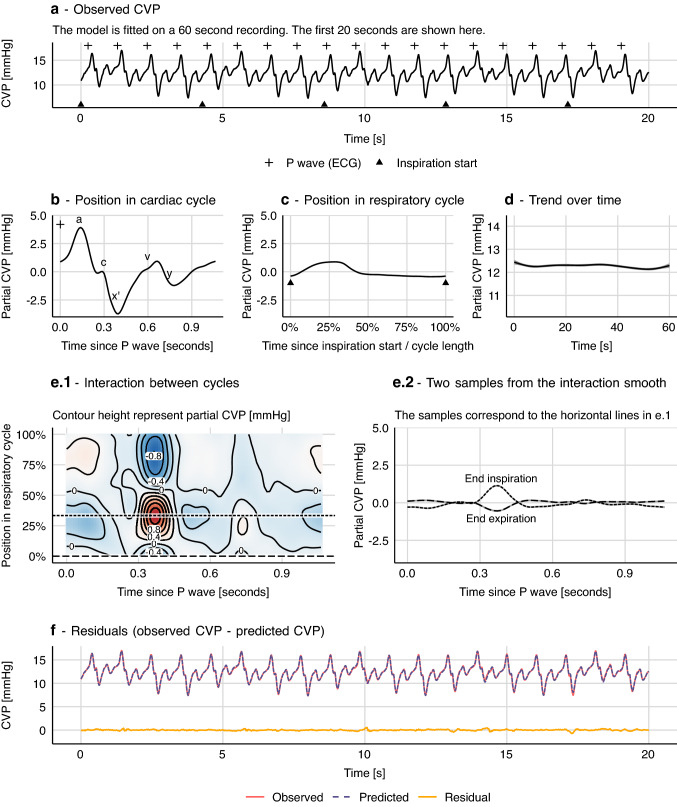
How a generalized additive model (GAM) can be fitted to a CVP waveform. **a** Each sample from a 125 Hz CVP waveform is represented with three predictor variables: position in cardiac cycle, position in respiratory cycle and time (seconds since sample start). A GAM is fitted giving the smooth functions **b** to **e** (the model constant (α) is added to the smooth function in **d**. **f** Model fit including residuals that are markedly reduced compared to the model without an interaction term, visualised in Fig. 4. Grey shades in panel b, c and e represent 95% confidence intervals (often too narrow to be visible)

$f\left({pos}_{cardiac},{pos}_{ventilation}\right)$ is a smooth function that represents the interaction between the cardiac and respiratory cycles. It is based on a non-cyclic spline in the x-direction (cardiac cycle) and a cyclic spline in the y-direction (respiratory cycle). It can be visualised as a surface (or more specifically, a cylinder, since it is cyclic in the Y direction), where the x-axis represents the cardiac cycle, the y-axis represents the respiratory cycle, and the z-axis represents the effect of the interaction on CVP (see Fig. 5).

**Fig. 6:**
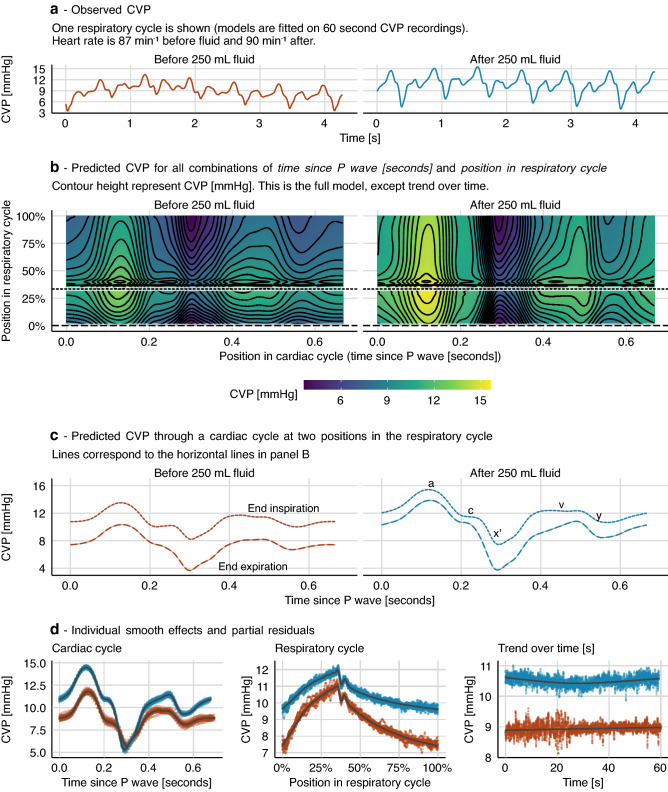
Comparison of sections of a generalized additive model (GAM). The model is fitted to two one-minute sections of central venous pressure (CVP) recordings. One section before a 250 ml fluid infusion and one after. A grey shade in panel **c** represents 95% confidence intervals (often too narrow to be visible). The constant terms ($\alpha$ and ${\beta }_{s}$) are included in all predictions (**b**, **c** and **d**)

To aid comprehension of the model—CVP as the interaction of two repeating cycles—we attach an animation of the model’s prediction, simultaneously on a time scale and projected onto a plane with cardiac cycle position and respiratory cycle position as independent variables (see Online Resource 2). The plane is equivalent to the contour plot in Fig. 6b, before 250 ml fluid.

#### Autocorrelation

Like other regression models, a GAM assumes that observations are independent, conditional on the model (i.e. that the residuals are independent). First, if there is some pattern remaining in the residuals, it is important to consider that the model may have underfitted the data (as in the example without an interaction term; shown in Fig. 4). But, even with an “optimal” fit, models of high resolution waveforms will likely have a high degree of autocorrelation in the residuals, as noise itself is often autocorrelated in these waveforms. To correct for this, we have included in the CVP models a first-order autoregressive model [AR(1)] for the residuals (see Online Resource 1 for details). Failure to deal with autocorrelation will give too narrow confidence intervals and can cause overfitting [20, 21].

#### How the CVP waveform changes after a fluid bolus

To illustrate the type of responses that can be estimated, we fitted a GAM to two one-minute sections of a CVP recording: the first section before administration of a 250 ml fluid bolus and the other after. Separate splines were fitted to each section:

$$CVP=\alpha +{\beta }_{s}+{f}_{s}\left({pos}_{cardiac}\right)+{f}_{s}\left({pos}_{ventilation}\right)+{f}_{s}\left({pos}_{cardiac},{pos}_{ventilation}\right)+{f}_{s}\left(time\right)+\epsilon ,$$

where ${\beta }_{s}$ is an additional constant, that is zero for the pre-fluid section, and ${f}_{s}$ is a spline for each section of data (before or after 250 ml fluid). This model also extends the previous model (Fig. 5) by using an *adaptive* smoothing spline to estimate ${f}_{s}\left({pos}_{ventilation}\right)$. An adaptive smoothing spline allows the spline’s smoothing parameter to vary across the range of the independent variable. This allows the spline to adapt to the sharp transition between inspiration and expiration, and to fit a subtle disturbance at the beginning of the expiration^2^ while remaining smooth in areas where there is no change in the effect of the independent variable on the response. The model is visualised in Fig. 6. We see that after fluid, this subject’s CVP varies more over a cardiac cycle, but less over a respiratory cycle, compared to before fluid. This is clearest in Fig. 6d. In Fig. 6c, we show the predicted CVP at end expiration and at end inspiration before and after fluid. This lets us compare how the interaction between the cardiac cycle and the respiratory cycle changes with fluid administration. The pressure during atrial contraction (*a* wave in Fig. 6c) increases with fluid, but the effect of ventilation on this pressure is lower after fluid. Another interesting difference is the shape of the *v* wave, representing the pressure in the right atrium before the tricuspid valve opening. Before fluid, the *v* wave has a flat peak, but after fluid, it increases gradually and reaches a higher pressure. This difference disappears at end-inspiration.

#### Dealing with signal noise

**Fig. 7:**
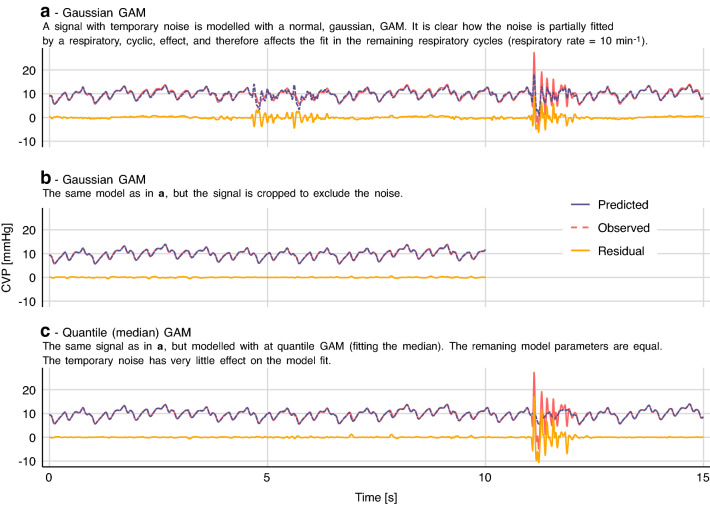
Quantile generalized additive models (QGAM) robustly fit medical signals with non-normal errors. The models correspond to the model shown in Fig. 5

In the examples above, we have fitted data on the assumption that errors (residuals) are normally distributed. In practice, extreme outliers are much more common than expected from a normal distribution. Most of the time, the measured signal (e.g. CVP) will reflect the true state with very little noise. However, temporary large deflections of the waveform are common (e.g. due to manipulation of transducer or tubing). Together, these two sources of noise give rise to errors that are both non-normal and heteroscedastic (with non-constant spread). If we try to fit this data with a model that assumes homoscedastic, normally distributed errors, we will likely encounter overfitting. This is illustrated in Fig. 7a, where the noise at 12 s is also predicted one respiratory cycle earlier—a least squares regression will prioritise being a little wrong twice over being doubly wrong once (since the errors are squared).

To remedy the problem with noise having a high impact on the fit, an effective approach is to fit the median of the signal with a *quantile* GAM. When fitting the median, there is no assumption about the conditional distribution of the dependent variable, and outliers (e.g. from noise) have a much lower impact on the model fit (see Fig. 7c). The *qgam* package by Fasiolo et al. extends *mgcv* to allow fitting quantile models [22].

## Discussion

In this methodological paper, we demonstrate how GAMs can be used as a flexible tool for modelling cyclic medical time series and waveforms. We give two heart–lung interaction examples: The first is a specific use-case: a robust calculation of pulse pressure variation from a time series of pulse pressure measurements. The second is a demonstration of how we can use a relatively simple model to fit the CVP waveform, with very little preconception of the shape of the waveform.

### Possible applications of GAMs

Currently, GAMs are research tools that may aid investigation of complex, yet deterministic patterns in medical time series and waveforms. Respiratory variation in hemodynamic variables is often just regarded as a potential source of error, sometimes dealt with by reporting only end-expiatory measurements. There may be clinically relevant information in the respiratory variation of measurements and GAMs give researchers a powerful tool for visualising and describing the effects of ventilation on their measurement of interest. It would be interesting to see GAMs like those demonstrated here for the CVP waveform and its changes during a respiratory cycle correlated to echocardiographic measurements like tricuspid annular plane systolic excursion (TAPSE) or other measures of right ventricular function. In particular, one hypothesis is that the *x’* descent and its dynamics during a respiratory cycle reflect right ventricular contraction against varying afterload [23]. Another CVP feature of interest is the *y* descent, whose magnitude is related to the rate of right ventricular filling during diastole. A large *y* descent has been proposed to indicate a *non*-fluid-responsive heart [24]. This hypothesis, and the respiratory variation in the *y* descent, could be further investigated using GAMs of CVP waveforms. CVP morphology has not had a prominent place in the scientific literature for decades, although venous return and mean systemic filling pressure are gaining more interest [25, 26]. The detailed dynamics of the CVP waveform during mechanical ventilation may reflect “upstream aspects” of venous return, mean systemic filling pressure and conditions for outflow of organs such as the kidneys. These might be elucidated by the diastolic parts of the CVP waveform.

A GAM of the arterial blood pressure waveform (and not just PPs) could give a more nuanced picture of the variation in left ventricular contraction.

As a clinical tool, estimation of PPV using a GAM could be implemented in a bedside monitor. The PPV could be presented along with a visualisation of the model fit (similar to Fig. 2c and d) for a clinician to decide if, e.g., a high PPV should be interpreted as noise or a true respiratory variation. Such interpretation, however, may require more than basic understanding of the physiologic determinants of PPV.

Another intriguing use case is that by Wyffels et al. They use a GAM to separate the seemingly random PPV from patients with atrial fibrillation into variation caused by ventilation and variation caused by the atrial fibrillation [4]. In this regard, both the respiratory component as well as the atrial fibrillation component may offer insights concerning fluid responsiveness, because blood pressure changes induced by filling time changes (induced by extrasystoles) have also predicted fluid responsiveness with acceptable accuracy in the intensive care unit [27, 28].

### Limitations

In the examples, we use synchronised data from both the ventilator and the bedside monitor. This is rarely available in data that is not recorded specifically to study heart–lung interactions. It is possible to fit these models if only the respiratory rate is known (by using the modulo operation of time over respiration length), though the phase of the respiratory effect will be arbitrary [4]. In many cases, the respiratory rate can be assessed by frequency analysis; fourier analysis for recordings with a constant sample rate (e.g. CVP) or Lomb-Scargle analysis for irregular time series (e.g. pulse pressure).

The models presented here assume that all respiratory cycles are equivalent. This requires deeply sedated, mechanically ventilated subjects. Therefore, the models presented here are most suitable in the setting of general anaesthesia. It is possible that the models could be extended to account for spontaneous ventilation efforts, e.g., by including esophageal- or airway pressure as independent variables in the model.

The CVP model uses a non-cyclic spline to model the effect of a cardiac cycle. We expect that the CVP at the end of one cardiac cycle continues smoothly into the following cycle, but this expectation is not enforced in our model. We cannot simply use a cyclic spline, as they require a fixed cycle length, while the cardiac cycle length varies with respiration. We could use the relative position in the cardiac cycle (from 0 to 1) as the independent variable in a cyclic spline, but this assumes that the cardiac cycle effect scales linearly with cardiac cycle length (i.e. if the cardiac cycle length is 10% longer, the time from, e.g., the ‘*a* peak’ to the ‘*v* peak’ should be 10% longer), which is not the case. Using non-cyclic splines to model the cardiac cycle gives the model some “unnecessary” degrees of freedom, and a better solution may exist.

It can be computationally expensive to fit GAMs, especially with large, high-resolution data sets and when interaction terms are introduced. The CVP model used in Fig. 5 takes ~ 60 s to fit on a modern laptop, currently making it infeasible for real time implementation. The quantile model used in Fig. 7 takes ~ 300 s for just 15 s of signal (1875 samples). The PP model in Fig. 2 takes only ~ 30 ms.

## Conclusion

Generalized additive models provide an intuitive and flexible approach to modelling the repeating signals common to medical monitoring data. We hope researchers will use this introduction as a starting point for including GAMs in their data analyses. Both to answer specific research questions, and as a tool to explore and visualise the cardiac effects and respiratory effects on hemodynamic measurements and the effect of heart–lung interactions.

## Recommended reading

*Generalized Additive Models, An Introduction with R* by Simon Wood [29].

GAMs in R by Noam Ross, A Free, Interactive Course using mgcv (https://noamross.github.io/gams-in-r-course/).

*Modelling Palaeoecological Time Series Using Generalised Additive Models* [20]. An introduction to GAMs with a more detailed description of the statistical considerations related to modelling time series and the inferences that can be drawn from the models.

*Hierarchical generalized additive models in ecology: an introduction with mgcv* [30]. The present paper only describes models fitted to data from one individual. A relevant next step is to fit one model across multiple individuals.

## Supplementary Information

Below is the link to the electronic supplementary material.

Supplementary file1 (PDF 5077 KB)

Supplementary file2 (MP4 6782 KB)

## Footnotes

1 Alternatively, QRS-complexes from the ECG could be used to mark the time of each heart beat. Pulse transit time is around 200 ms and it varies approximately 10–20 ms with ventilation [16]. Therefore, using QRS-complexes to time each heart beat would create a slight leftwards phase shift of the respiratory cycle smooth (Fig. 2c) and a probably unnoticeable effect of the variation in pulse transit time. For patients with cardiac arrhythmia, using QRS-complexes could aid the analysis. Both because it may be difficult to identify individual heart beats from the ABP waveform alone, and because pulse transit time might vary significantly between beats.

2 The small disturbance at the beginning of the inspiration corresponds to the closing of the ventilator solenoid valve at end-inspiration. The sudden drop in pressure makes the ventilator tubing move and disturb the adjacent CVP line. It is most visible in Fig. 6d, but can also be recognized in the residuals in Fig. 5f.
