# Supplementary material for: Using generalized additive models to decompose time series and waveforms, and dissect heart–lung interaction physiology
Source: J Clin Monit Comput. 2022 Jun 13;37(1):165–77. doi: 10.1007/s10877-022-00873-7 (PMC9852126; doi:10.1007/s10877-022-00873-7)
Supplement: Supplementary file 1 — Supplementary file1 (PDF 5077 KB) [file 10877_2022_873_MOESM1_ESM.pdf]

# A practical guide to fitting GAMs to medical monitoring data with *mgcv* and R

**Johannes Enevoldsen**,
Department of Clinical Medicine, Aarhus University and Department of Anaesthesiology & Intensive Care, Aarhus University Hospital,
Aarhus, Denmark
Email: enevoldsen@clin.au.dk

**Gavin L Simpson**,
Department of Animal Science, Aarhus University,
Tjele, Denmark

**Simon T Vistisen**,
Department of Clinical Medicine, Aarhus University and Department of Anaesthesiology & Intensive Care, Aarhus University Hospital,
Aarhus, Denmark

**Abstract**

Supplement to the article: **Using Generalized Additive Models to Decompose Time Series and Waveforms, and Dissect Heart-lung Interaction Physiology** in **Journal of Clinical Monitoring And Computing**. The document examplifies how to fit and work with generalized additive models (GAMs) in R using *mgcv*.

# Contents


**1 Data and source code** **2**

**2 Packages** **2**

**3 Pulse pressure** **3**

3.1 Load data . . . 3

3.2 Detect heart beats . . . 4

3.3 Fit and visualise the GAM . . . 8

3.4 Calculate pulse pressure variation . . . 11

**4 Central venous pressure** **13**

4.1 Load data . . . 13

4.2 Fit and visualise the GAM . . . 17

4.3 Adaptive smoothness . . . 19

4.4 Autocorrelated residuals . . . 21

4.5 The full model with two sections of data: before and after fluid . . . 24

4.6 Other common challenges . . . 27

**5 Session info** **30**


# 1 Data and source code

Sample data and the source code for this document is available at https://doi.org/10.5281/zenodo.6375221.

# 2 Packages

Packages used in these examples can be installed from CRAN using `install.packages("package name")`.

```
library(mgcv) # fit GAMs
library(gratia) # visualise and work with GAMs
library(dplyr) # work with data frames
library(ggplot2) # plots
library(patchwork) # combine plots

# Packages only used for heart beat detection (dependencies of `find_abp_beats()`)
# install.packages("RcppRoll")
# install.packages("purrr")
```

```
theme_set(theme_minimal()) # Change the default plotting theme

# Data preparation functions. Available in code repository.
source("functions.R")
```

# 3 Pulse pressure

The first example corresponds to the model shown in Fig. 2 in the paper. The aim is to model variation in pulse pressure using information about the respiratory cycle. The model is then used to estimate pulse pressure variation (PPV).

## 3.1 Load data

We use a 30 second recording of arterial blood pressure (ABP). The sample data is structured as a list. It includes ABP `sample_pp$abp` and the start time of each inspiration `sample_pp$insp_start`.

```
sample_pp <- readRDS("sample_PP.RDS")
```

The ABP is stored as a data frame with a time column and a pressure column. The time is in seconds (sample rate 125 Hz) and the pressure unit is mmHg.

```
head(sample_pp$abp)
```

```
## # A tibble: 6 x 2
##      time   ABP
##     <dbl> <dbl>
## 1 0.00588  60.5
## 2 0.0139   60.8
## 3 0.0219   61.1
## 4 0.0299   61.2
## 5 0.0379   61.4
## 6 0.0459   61.6
```

The timing of inspirations is a data frame with a time column that indicates when a new inspiration starts. These time points are recorded from a Draeger ventilator, but could be determined from any recorded airway pressure waveform.

```
head(sample_pp$insp_start)
```

```
## # A tibble: 6 x 2
##    time label
##   <dbl> <chr>
## 1  2.49 Insp start
## 2  6.25 Insp start
```

```
## 3  9.98 Insp start
## 4 13.7  Insp start
## 5 17.5  Insp start
## 6 21.2  Insp start
```

We can visualise the two data frames together.

```
abp_plot <- ggplot(sample_pp$abp, aes(time, ABP)) +
  geom_line() +
  geom_vline(aes(xintercept = time), color = "red",
             data = sample_pp$insp_start)

abp_plot
```

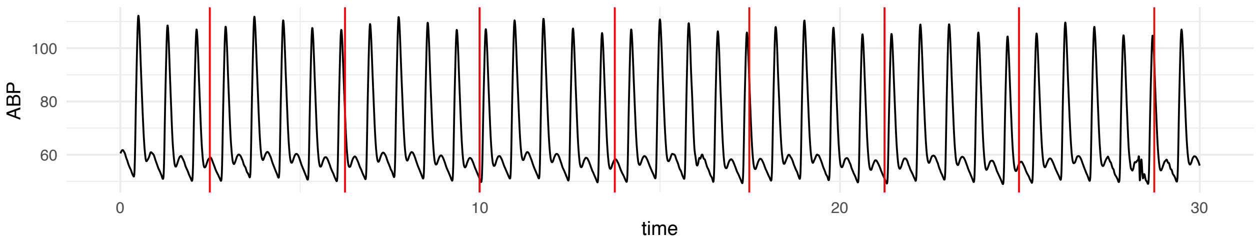


Figure 1: Arterial blood pressure (ABP). Red lines indicate inspiration start.

### 3.2 Detect heart beats

We now detect individual heart beats from the ABP waveform. A function, `find_abp_beats()`, is shared in the file `functions.R`. It takes an ABP waveform and returns a data frame of individual beats. The data frame contains the following columns (plus a few more that we do not use in this example):

- `time`: timing of the diastole (negative peak) [seconds]. This marks the beginning of a beat
- `dia`: diastolic pressure [mmHg]
- `time_systole`: timing of the systole (positive peak) [seconds]
- `sys`: the following systolic pressure [mmHg]
- `PP`: pulse pressure (`sys - dia`) [mmHg]
- `beat_len`: length of the beat (`time - lead(time)`) [seconds], which roughly corresponds to an RR interval

```
beats <- find_abp_beats(sample_pp$abp)
head(beats)
```

```
## # A tibble: 6 x 10
##    time   dia time_systole   sys    PP beat_len dia_pos sys_pos .noise_wiggline~
##   <dbl> <dbl>        <dbl> <dbl> <dbl>    <dbl>   <dbl>   <dbl>            <dbl>
## 1 0.366  51.8        0.494  112.  60.4    0.808      46      62             17.1
```

```
## 2 1.17   50.9        1.31   108.  57.6    0.808     147     164              16.2
## 3 1.98   50.2        2.11   107   56.8    0.808     248     264              15.7
## 4 2.79   50.2        2.92   108.  57.9    0.8       349     365              15.6
## 5 3.59   51.5        3.72   112.  60.3    0.8       449     465              16.5
## 6 4.39   51.4        4.52   110.  59.1    0.808     549     565              16.6
## # ... with 1 more variable: .noise_pos_after_sys <dbl>
```

We can add this information to the previous plot.

```
abp_plot +
  geom_point(aes(x = time,
                 y = dia,
                 colour = "diastole"),
             data = beats) +
  geom_point(aes(x = time_systole,
                 y = sys,
                 colour = "systole"),
             data = beats) +
  # Place systole above diastole in legend
  scale_color_discrete(limits = c("systole", "diastole"))
```

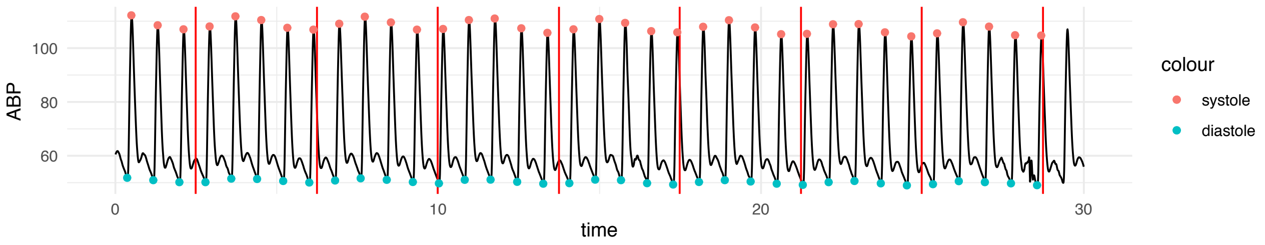


Figure 2: Beats are detected using 'find_abp_beats()'.

For this model, we are only interested in the pulse pressure (PP = systolic pressure - diastolic pressure) of each heart beat.

```
pp_plot <- ggplot(beats, aes(time, PP)) +
  geom_line() +
  geom_point() +
  geom_vline(aes(xintercept = time), color = "red",
             data = sample_pp$insp_start)

pp_plot
```

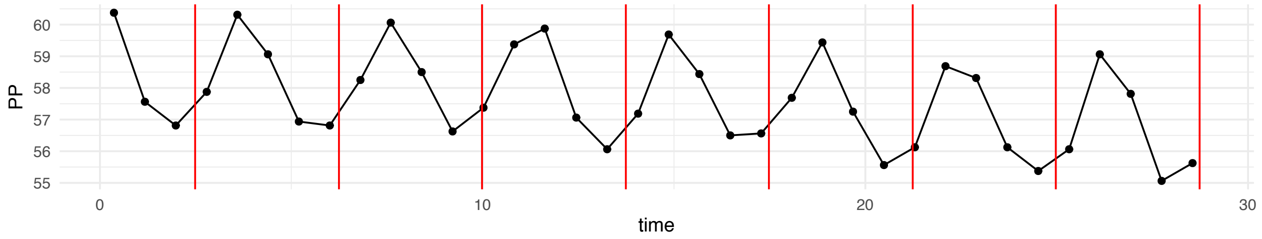


Figure 3: Pulse pressure (PP) of each heart beat.

Before we can fit the model, we need to calculate the position of each beat in the respiratory cycle. `functions.R` contain `add_time_since_event()`, that takes a data frame with a time column (here `beats`) and a vector of times corresponding to some event (here the timing of each inspiration start: `sample_pp$insp_start$time`) and returns the data frame with new columns indicating the timing of each observation (beat) relative to the most recent event (inspiration start). The new columns are:

- `insp_index`: time since the latest inspiration start [seconds]
- `insp_n`: respiratory cycle number
- `insp_cycle_len`: length of the respiratory cycle [seconds]
- `insp_rel_index`: the relative position of the beat in the respiratory cycle (`insp_index` / `insp_cycle_len`)

```
beats_indexed <- add_time_since_event(beats,
                                      time_events = sample_pp$insp_start$time,
                                      prefix = "insp") %>%
  # the first beats are earlier than the first inspiration and
  # therefore have `insp_rel_index = NA`. We remove these.
  na.omit()

# Relocate time and the four newly added columns.
head(beats_indexed %>% relocate(time, starts_with("insp_")))
```

```
## # A tibble: 6 x 14
##    time insp_index insp_n insp_cycle_len insp_rel_index   dia time_systole   sys
##   <dbl>      <dbl>  <int>          <dbl>          <dbl> <dbl>        <dbl> <dbl>
## 1  2.79      0.303      1           3.76         0.0805  50.2         2.92  108.
## 2  3.59      1.10       1           3.76         0.293   51.5         3.72  112.
## 3  4.39      1.90       1           3.76         0.506   51.4         4.52  110.
## 4  5.20      2.71       1           3.76         0.721   50.6         5.33  108.
## 5  6.01      3.52       1           3.76         0.936   50.1         6.13  107.
## 6  6.81      0.560      2           3.74         0.150   50.8         6.93  109.
## # ... with 6 more variables: PP <dbl>, beat_len <dbl>, dia_pos <dbl>,
## #   sys_pos <dbl>, .noise_wiggliness <dbl>, .noise_pos_after_sys <dbl>
```

This lets us show each beat by its position in the respiratory cycle.

```
pp_plot_color <- ggplot(beats_indexed, aes(time, PP)) +
  geom_line() +
  # insp_n is a unique (consecutive) number for each respiratory cycle
  geom_point(aes(color = as.factor(insp_n)), show.legend = FALSE) +
  geom_vline(aes(xintercept = time), color = "red",
             data = sample_pp$insp_start) +
  labs(title = "Pulse pressure",
       subtitle = "by time. Color indicate respiratory cycle (`insp_n`)")

pp_insp_plot <- ggplot(beats_indexed,
                       aes(
                         insp_rel_index,
                         PP,
                         group = as.factor(insp_n),
                         color = as.factor(insp_n)
                         )
                       ) +
  geom_line(alpha = 0.3, show.legend = FALSE) +
  geom_point(show.legend = FALSE) +
  labs(subtitle = "by position in the respiratory cycle")

pp_plot_color + pp_insp_plot + plot_layout(widths = c(2,1))
```

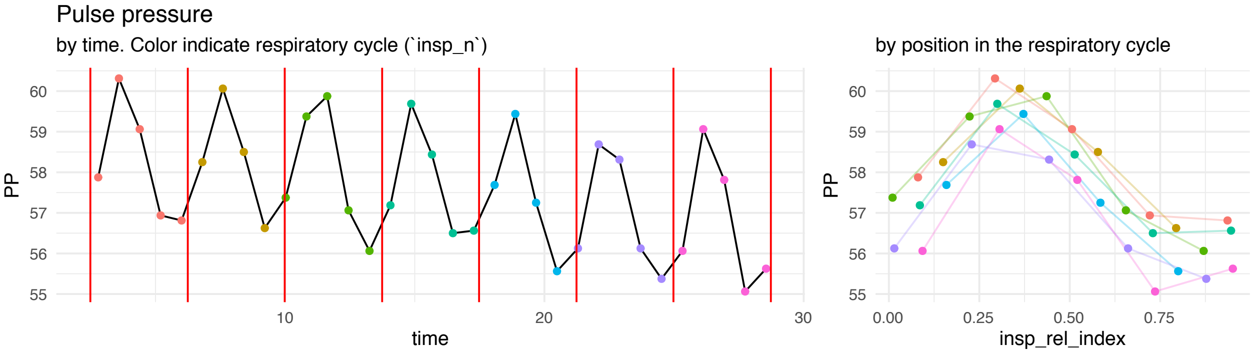


Figure 4: Pulse pressure indexed to the respiratory cycle.

We now have the variables we need to fit the model. For clarity, we select only the variables we need for the model.

```
PP_data <- select(beats_indexed, PP, time, insp_rel_index)

head(PP_data)
```

```
## # A tibble: 6 x 3
##      PP  time insp_rel_index
##   <dbl> <dbl>          <dbl>
## 1  57.9  2.79         0.0805
```

```
## 2  60.3  3.59         0.293
## 3  59.1  4.39         0.506
## 4  56.9  5.20         0.721
## 5  56.8  6.01         0.936
## 6  58.2  6.81         0.150
```

### 3.3 Fit and visualise the GAM

To fit the model, we use the `gam()` function from `mgcv`.

```
PP_gam <- gam(
  # The first parameter to the gam() function is the model specification,
  # supplied using formula notation:

  # Left of the tilde (~) is our dependent variable PP
  PP ~
    # Right of the tilde is our independent variables.

    # Define a smooth function of insp_rel_index.
    s(insp_rel_index,
      k = 15, # 15 knots.
      bs = "cc" # The basis is a cyclic cubic spline
      ) +
    # Define a smooth function of time
    s(time,
      bs = "cr" # The basis is a natural cubic spline.
      # default k is 10. This will be fine here.
      ),

  # We can specify the positions of the knots for each smooth.
  # If only two knots are specified for a cyclic spline, these will
  # set the positions of the limiting knot(s). The remaining knots will
  # be positioned automatically (for cubic splines, the default position
  # is at quantiles of the independent variable).
  knots = list(insp_rel_index = c(0,1)),

  # We use restricted maximum likelihood (REML) to fit the optimal smoothing parameter.
  # This is often the best choice, but not the default.
  method = "REML",

  data = PP_data
)
```

Now, plot the model using `gratia::draw()` (or `plot()`). Adding partial residuals is a simple way to visualise how well the model fits observed data and to identify systematic errors. Partial residuals are the model residuals + the specific smooth effect in the plot.

```
draw(PP_gam,
     residuals = TRUE)
```

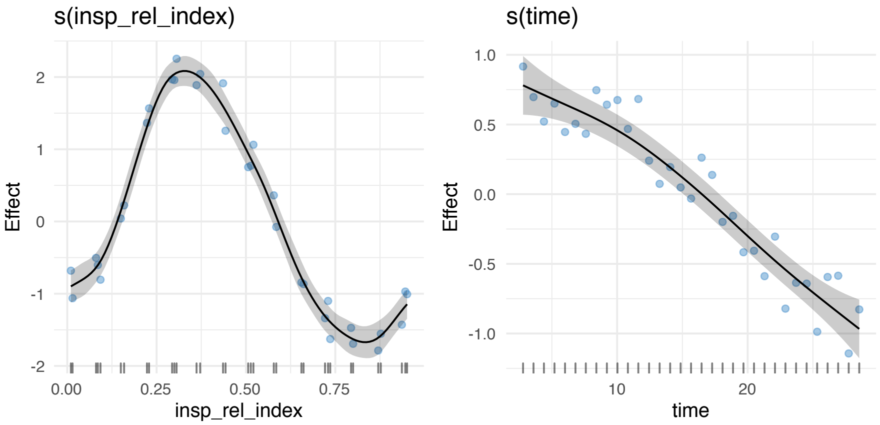


Figure 5: Smooth effects of the pulse pressure GAM.

In addition to the two smooth effects, the model also has a constant/intercept, which is not visualised in the plots above. In this model, the intercept is the mean PP.

```
coef(PP_gam)[1]
```

```
## (Intercept)
##    57.59848
```

Predictions (or fit) can be calculated as

$$\hat{PP} = \alpha + s(\text{insp\_rel\_index}) + s(\text{time}),$$

where $\alpha$ is the model intercept.

We can use `predict()` to calculate model predictions.[1] If we only pass the GAM, it will make predictions using the observed independent variables we used to fit the model. We can add these predictions as a new column to our original dataset.

```
PP_pred <- mutate(PP_data, pred = predict(PP_gam))

ggplot(PP_pred, aes(x=time)) +
  geom_line(aes(y=PP, color = "Observed")) +
  geom_point(aes(y=PP, color = "Observed")) +
  geom_point(aes(y=pred, color = "Predicted"))
```

[1]When `predict()` is called on a GAM (an object of class “gam”), the specific method `predict.gam()` is used. See `?predict.gam()` for help.

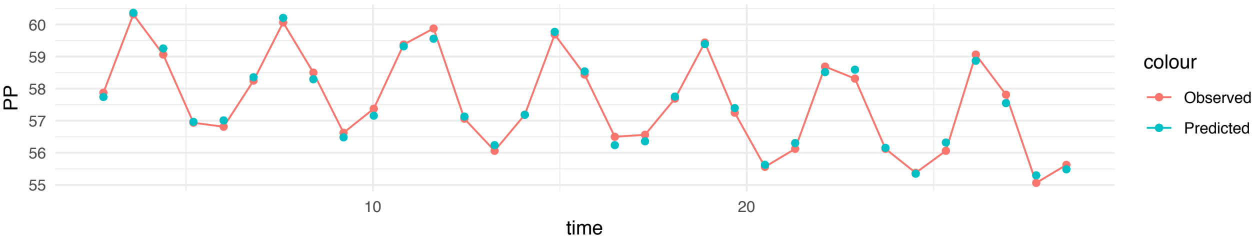


Figure 6: Observed and predicted pulse pressure.

We can use the `newdata` parameter in `predict()` to interpolate predictions between our observations (it rarely makes sense to extrapolate a spline fit).

```
PP_newdata <- tibble(
  # create 200 points from 0 to 30 to make the prediction visually smooth
  time = seq(0, 30, length.out = 200)) %>%
  # index each new time to our existing vector of inspiration times
  add_time_since_event(sample_pp$insp_start$time, prefix = "insp") %>%
  na.omit()

PP_interpolate <- bind_cols(
  PP_newdata,
  predict(PP_gam,
          newdata = PP_newdata,
          # in addition to the predictions (fit) we can also return the standard error
          # (se.fit) for each prediction. This makes predict return a named list,
          # that we can simply bind as columns to our data frame.
          se.fit = TRUE)
)

ggplot(PP_interpolate, aes(x = time)) +
  geom_ribbon(aes(ymin = fit - 1.96*se.fit,
                  ymax = fit + 1.96*se.fit,
                  fill = "Predicted (95% CI)")) +
  geom_line(aes(y = fit)) +
  geom_point(aes(y=PP, color = "Observed"), data = PP_data) +
  scale_fill_manual(values = "skyblue") +
  labs(x = "PP")
```

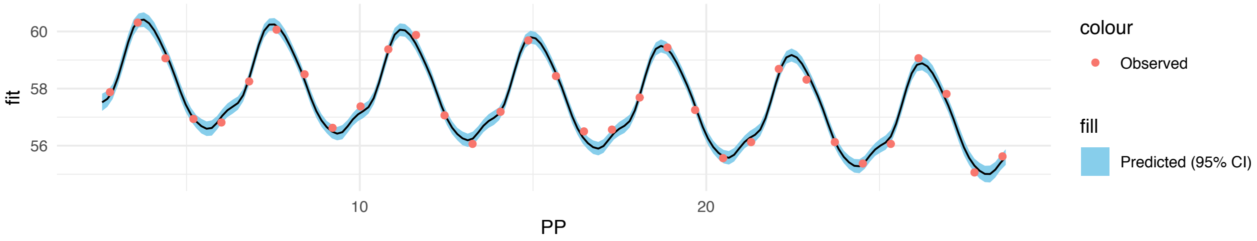


Figure 7: Observed and predicted pulse pressure, including predictions between observations.

## 3.4 Calculate pulse pressure variation

We can calculate pulse pressure variation (PPV) from this model using the formula

$$PPV = \frac{maximum(s(\text{insp\_rel\_index})) - minimum(s(\text{insp\_rel\_index}))}{\alpha},$$

where $\alpha$ is the model constant (intercept). To find the extrema of the smooth, we can generate a grid of predictions using only the one smooth term `s(insp_rel_index)`. We could use `predict(type="terms")` but `gratia::smooth_estimates()` conveniently returns the values of the smooth over the original range of the independent variable (here `insp_rel_index`).

```
insp_rel_index_smooth <- smooth_estimates(PP_gam,
                                          smooth = "s(insp_rel_index)",
                                          n=100)
min_PP <- min(insp_rel_index_smooth$est)
max_PP <- max(insp_rel_index_smooth$est)
intercept_PP <- coef(PP_gam)[1]
PPV_est <- (max_PP - min_PP) / intercept_PP

sprintf("PPV is %.1f%%", PPV_est*100)
```

```
## [1] "PPV is 6.5%"
```

This PPV is an estimate from the model. We should also report the uncertainty. `mgcv` lets us sample parameters from the posterior distribution of the model (similarly to posterior sampling from a Bayesian model; see Gavin Simpson's answer on StackOverflow for an in-depth example of how this can be done). Here we need posterior samples of a smooth, and, conveniently, `gratia::smooth_samples()` does just that. For each sampled smooth, we can calculate PPV, and use the many samples of PPVs to calculate a confidence interval for PPV (this approach neglects that the model intercept is also an estimate, but since the standard error for the intercept is *very* small, this has negligible effect on the width of the confidence interval).

```
set.seed(1)
# Sample 5000 smooths
insp_smooth_samples <- smooth_samples(PP_gam, term = "s(insp_rel_index)", n = 5000)

# Draw the first 50 samples.
ggplot(insp_smooth_samples %>% filter(draw <= 50), aes(.x1, value, group = draw)) +
  geom_line(alpha = 0.1) +
  labs(x="insp_rel_index")
```

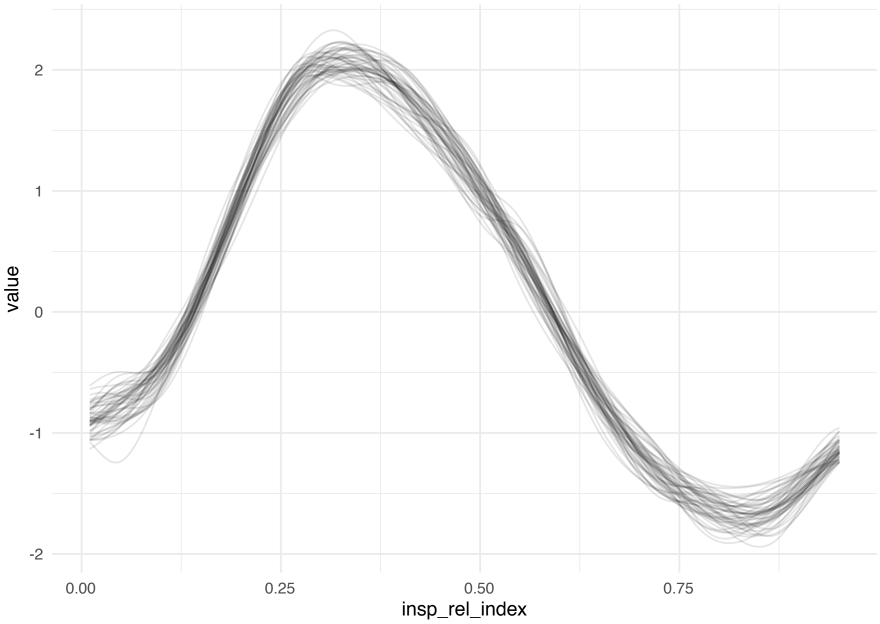


Figure 8: Plot of 50 of the 5000 sampled smooths from the posterior distribution of the respiratory cycle smooth ‘s(insp_rel_index)‘.

```
# A function that returns PPV given a smooth and an intercept
calc_PPV <- function(smooth, intercept) {
  min_PP <- min(smooth)
  max_PP <- max(smooth)
  (max_PP - min_PP) / unname(intercept)
}

PPV_samples <- insp_smooth_samples$value %>%
  split(insp_smooth_samples$draw) %>%
  sapply(calc_PPV, intercept = coef(PP_gam)[1])

PPV_95 <- quantile(PPV_samples, probs = c(0.025, 0.975))

ggplot(data.frame(PPV = PPV_samples), aes(x=PPV)) +
  geom_histogram() +
  geom_vline(aes(xintercept = PPV_est, color = "Estimate"),
             data = data.frame()) +
  geom_vline(aes(xintercept = PPV_95, color = "95% confidence interval"),
             data = data.frame()) +
  scale_x_continuous(labels = scales::label_percent())
```

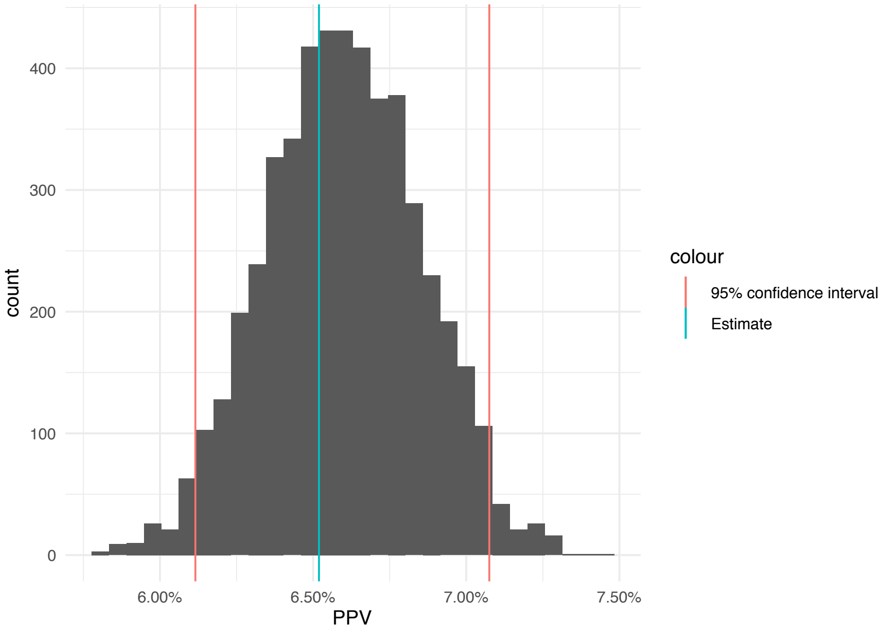


Figure 9: Histogram of PPVs calculated from each of the 5000 sampled smooths from the posterior distribution of the respiratory cycle smooth ‘s(insp_rel_index)‘.

# 4 Central venous pressure

These examples correspond to the models shown in Fig. 5 and 6 in the paper.

## 4.1 Load data

We use a section of a recording of central venous pressure (CVP) `sample_cvp$cvp`. The sample data is structured as a list. In addition to CVP, it also includes the start time of each inspiration `sample_cvp$insp_start` and of each QRS-complex `sample_cvp$qrs` and the interval in which 250 ml fluid is administered (`sample_cvp$fluid_start` to `sample_cvp$fluid_end`).

```
sample_cvp <- readRDS("sample_CVP.RDS")
```

The CVP is stored as a data frame with a time column and a pressure column. The time is in seconds, the sample rate is 125 Hz and the pressure unit is mmHg.

```
head(sample_cvp$cvp)
```

```
## # A tibble: 6 x 2
##      time   CVP
##     <dbl> <dbl>
## 1 0.00432  9.44
## 2 0.0123   9.44
## 3 0.0203   9.56
```

```
## 4 0.0283   9.62
## 5 0.0363   9.75
## 6 0.0443   9.88
```

```
plot_cvp_full <- ggplot(sample_cvp$cvp, aes(time, CVP)) +
  annotate("rect", xmin = sample_cvp$fluid_start, xmax = sample_cvp$fluid_end,
           ymin = -Inf, ymax = Inf,
           fill = alpha("blue", 0.4)) +
  annotate("rect", xmin = sample_cvp$fluid_start-30, xmax = sample_cvp$fluid_start,
           ymin = -Inf, ymax = Inf,
           fill = alpha("green", 0.4)) +
  geom_line() +
  labs(title = "Full sample",
       subtitle = "Blue area: administration of 250 ml fluid
Green area: section used to fit GAM in the first example")

plot_cvp_short <- ggplot(sample_cvp$cvp, aes(time, CVP)) +
  geom_line() +
  coord_cartesian(xlim = c(sample_cvp$fluid_start-30, sample_cvp$fluid_start)) +
  geom_vline(aes(xintercept = time), color = "red",
            data = sample_cvp$insp_start) +
  geom_vline(aes(xintercept = time), color = "blue",
            data = sample_cvp$qrs) +
  labs(title = "Green area",
       subtitle = "Blue lines: QRS-complexes
Red lines: inspiration start")

plot_cvp_full/plot_cvp_short
```

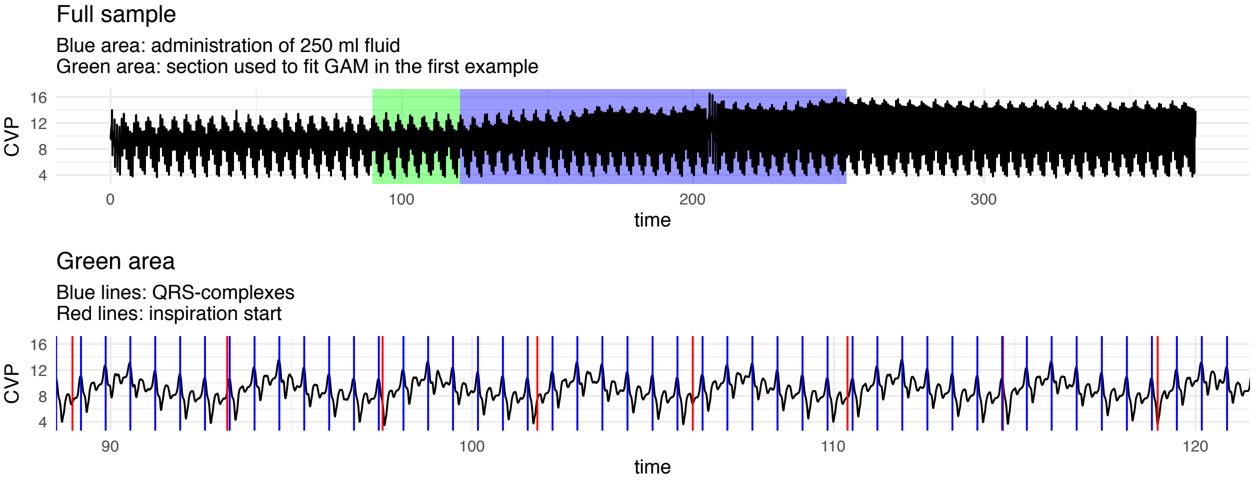


Figure 10: Sample data for CVP model.

We want to fit the model:

$$CVP = \alpha + f(pos_{cardiac}) + f(pos_{ventilation}) + f(pos_{cardiac},\ pos_{ventilation}) + f(t_{total}) + \epsilon.$$

First, we need to calculate each CVP sample's position in both the cardiac and respiratory cycles. For the respiratory cycle, we will fit a cyclic spline based on the relative position (as in the pulse pressure example above). For the cardiac cycle, we cannot simply use a cyclic spline, as the cycles vary in length. We could use a cyclic spline based on the relative position in the cardiac cycle of a CVP sample, but that would assume that the CVP waveform of a long cardiac cycle is simply a stretched version of a short cycle. Instead we assume that the cardiac cycle effect depends on the time since the P wave (initiation of atrial contraction), without constraining it to be cyclic. Instead of trying to detect P waves from the ECG, we assume that they appear a constant interval before the QRS complex.

In the sample data, QRS complexes have already been detected from `sample_cvp$ecg`. This was done with `rsleep::detect_rpeaks()`.

To find the PQ interval, we align 30 seconds of ECG recording by the detected QRS complexes.

```
sample_cvp$ecg %>%
  add_time_since_event(sample_cvp$qrs$time-0.3, prefix = "before_qrs") %>%
  na.omit() %>%
  filter(time < 30) %>%
  ggplot(aes(before_qrs_index-0.3, ECG_II, group = before_qrs_n)) +
  geom_line(alpha = 0.3) +
  scale_x_continuous(breaks = seq(-0.2, 0.4, by = 0.05)) +
  labs(x = "Time - QRS-time [seconds]")
```

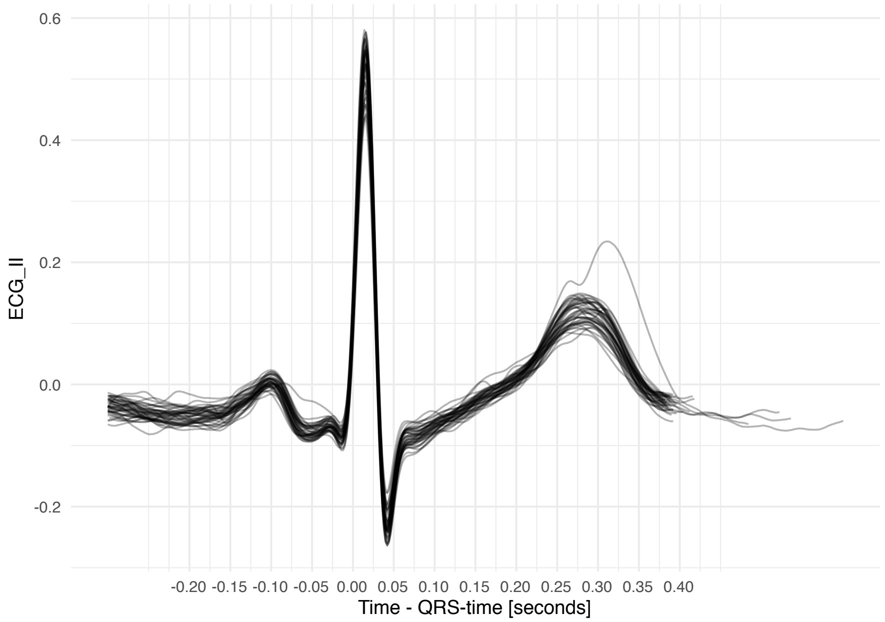


Figure 11: ECG recording (30 seconds) aligned by QRS complexes.

We can see that the P wave starts ~150 ms before the QRS complex.

In this example we will fit a GAM to the last 30 seconds before fluid administration starts (the green area in Figure 10). We add each sample's position in both the cardiac cycle (starting at the P wave) and the respiratory cycle, and filter the data to the relevant section.

```
PQ_interval <- 0.150 #seconds

cvp_df <- sample_cvp$cvp %>%
  # QRS time - PQ interval = P wave time.
  add_time_since_event(sample_cvp$qrs$time - PQ_interval, prefix = "P_wave") %>%
  add_time_since_event(sample_cvp$insp_start$time, prefix = "insp")

# Select a 30 second sample before fluid administration.
cvp_df_30 <- filter(cvp_df,
                    between(time, sample_cvp$fluid_start-30, sample_cvp$fluid_start))

head(cvp_df_30)
```

```
## # A tibble: 6 x 10
##    time   CVP P_wave_index P_wave_n P_wave_cycle_len P_wave_rel_index insp_index
##   <dbl> <dbl>        <dbl>    <int>            <dbl>            <dbl>      <dbl>
## 1  90.0  8.56        0.290      130            0.682            0.425       1.06
## 2  90.0  8.12        0.298      130            0.682            0.437       1.07
## 3  90.0  7.81        0.306      130            0.682            0.449       1.07
## 4  90.0  7.75        0.314      130            0.682            0.460       1.08
## 5  90.0  7.88        0.322      130            0.682            0.472       1.09
## 6  90.0  8.12        0.330      130            0.682            0.484       1.10
## # ... with 3 more variables: insp_n <int>, insp_cycle_len <dbl>,
## #   insp_rel_index <dbl>
```

In the above table, `P_wave_index` is the position in the cardiac cycle (seconds since latest P wave) and `insp_rel_index` is the position in the respiratory cycle (time since inspiration start relative to length of the respiratory cycle).

Before fitting the model, we can visualise (individually) the three effects we subsequently want to model (collectively): time, respiratory cycle and heart cycle.

```
cvp_time <- ggplot(cvp_df_30, aes(time, CVP)) +
  geom_line() +

  labs(title="Time")

cvp_insp <- ggplot(cvp_df_30, aes(insp_rel_index, CVP, group = insp_n)) +
  geom_line() +
  labs(title="Respiratory cycle (relative)")

cvp_p <- ggplot(cvp_df_30, aes(P_wave_index, CVP, group = P_wave_n)) +
  geom_line() +
  labs(title="Cardiac cycle (seconds since P wave)")
```

```
cvp_time / (cvp_insp + cvp_p)
```

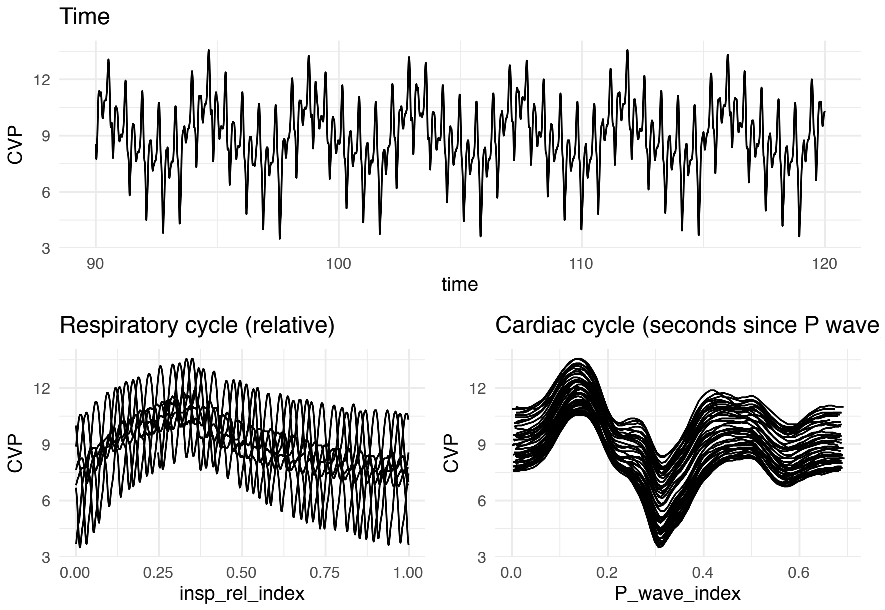


Figure 12: Different visualizations of the data used for the CVP model.

## 4.2 Fit and visualise the GAM

Now, we are ready to fit the model. This time we use `bam()` which is like `gam()`, but optimised for large datasets. First, we fit the model without correcting for autocorrelation of the residuals. A later section will demonstrate how to correct for autocorrelation.

```
gam_cvp <- bam(
      CVP ~
        s(P_wave_index, bs = "cr", k = 40) +

        s(insp_rel_index, bs = "cc", k = 30) +

        # We create the interaction smooth with ti() rather than te() because the main
        # effects, s(P_wave_index) and s(insp_rel_index), are also present in the model.
        # Separating the interaction from the main effects, allows mgcv to fit it with
        # separate smoothing parameters.
        # 40 x 30 knots makes a highly flexible plane, that will often overfit the data
        # (especially with a sample of just 30 seconds).
        # We can reduce overfitting either by fixing the smoothing parameters, for the
        # interaction smooth (with the `sp` parameter), or by using `bam`s `gamma`
        # parameter to increase smoothness for entire model.
        ti(
          P_wave_index,
          insp_rel_index,
```

```r
        bs = c("cr", "cc"),
        k = c(40, 30)
      ) +
      s(time, bs = "cr"), # If the detrending smooth captures some of the
      # other effects (respiratory or cardiac) it may be necessary to set a high
      # fixed smoothing parameter.

    knots = list(insp_rel_index = c(0, 1)),
    gamma = 5, # for a 125 Hz CVP waveform, `gamma = 5` seems to work well.
    data = cvp_df_30
  )
```

Visualise the smooth effects of the model.

```r
gratia::draw(gam_cvp, residuals = TRUE, rug = FALSE)
```

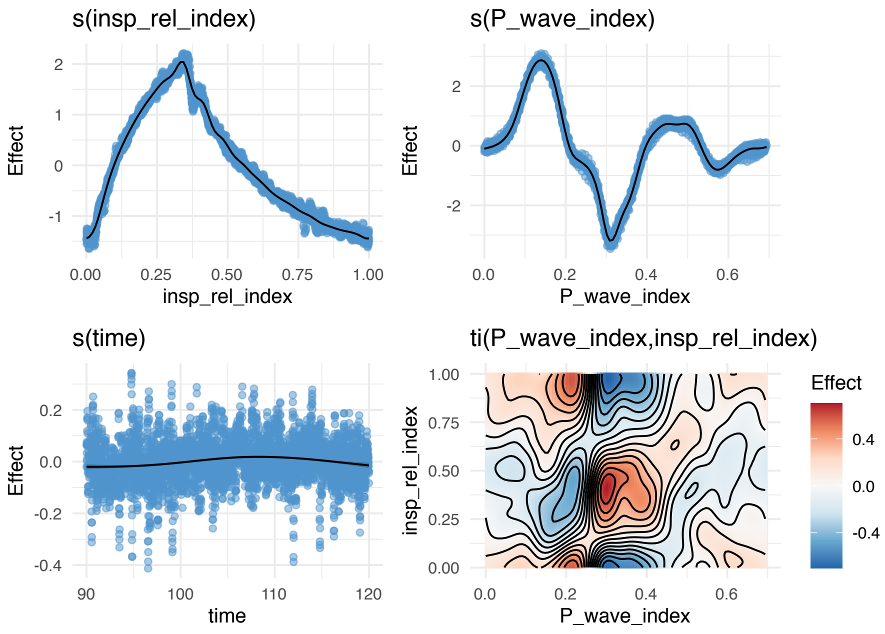


Figure 13: Smooth effects of the CVP GAM.

The model intercept (the mean CVP) is not visualised in the plots above.

```r
coef(gam_cvp)[1]
```

```
## (Intercept)
##    8.951227
```

We can visualise how well our model fits the observed CVP.

```r
cvp_df_pred <- mutate(cvp_df_30,
                             pred = predict(gam_cvp),
                             resid = resid(gam_cvp))

cvp_df_pred %>%
  ggplot(aes(time, CVP)) +
  geom_line() +
  geom_line(aes(y = pred), color = "red") +
  geom_line(aes(y = resid), color = "orange")
```

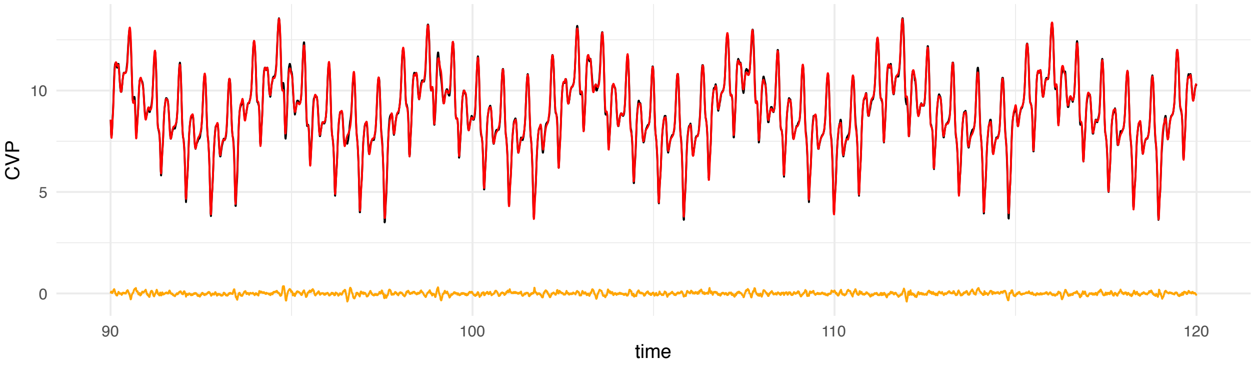


Figure 14: Observed, predicted and residual CVP

There is a small but clear pattern in the residuals one third into each respiratory cycle (most visible in the `s(insp_rel_index)` smooth in Figure 13). This corresponds to the closing of the ventilator solenoid valve at end-inspiration. The sudden drop in pressure makes the ventilator tubing move and disturb the adjacent CVP line. In the section below, we extend the respiratory cycle smooth to fit this residual signal.

### 4.3 Adaptive smoothness

The respiratory cycle effect does not have a constant smoothness. It has a sharp drop at start-expiration. In addition, this sample recording has a quick disturbance at end-inspiration, as described in the section above.

There are different ways to allow a change in smoothness across a spline. One is transformation of the independent variable (here `insp_rel_index`) to "stretch" the section with high wiggliness. This is computationally efficient, but requires the change in smoothness to be known *a priori*.

The method demonstrated here uses a spline with adaptive smoothness. This is an extension of a smoothing spline, where the smoothing parameter (wiggiliness penalty) is not constant but allowed to vary over the independent variable. The smoothing parameter is, itself, a spline estimated from data.

```r
gam_cvp_ad <- bam(
      CVP ~
        s(P_wave_index, bs = "cr", k = 40) +
```

```r
    # Adaptive smooth `bs = "ad"`, `m` is equivalent to `k`, but sets the number
    # knots for the spline that defines the adaptive penalty.
    # The `xt` parameter is used to specify the smoothing spline
    # (here a cyclic cubic spline).
    # We increase k to 60 to enable the spline to fit the extra wiggly section.
    s(insp_rel_index, bs = "ad", k = 60, m = 5, xt = list(bs = "cc")) +

    # The interaction smooth does not have adaptive smoothness.
    ti(
      P_wave_index,
      insp_rel_index,
      bs = c("cr", "cc"),
      k = c(40, 30)
    ) +
    s(time, bs = "cr"),
  knots = list(insp_rel_index = c(0,1)),
  data = cvp_df_30,
  gamma = 5
)
```

```r
gratia::draw(gam_cvp_ad, residuals = TRUE, rug = FALSE)
```

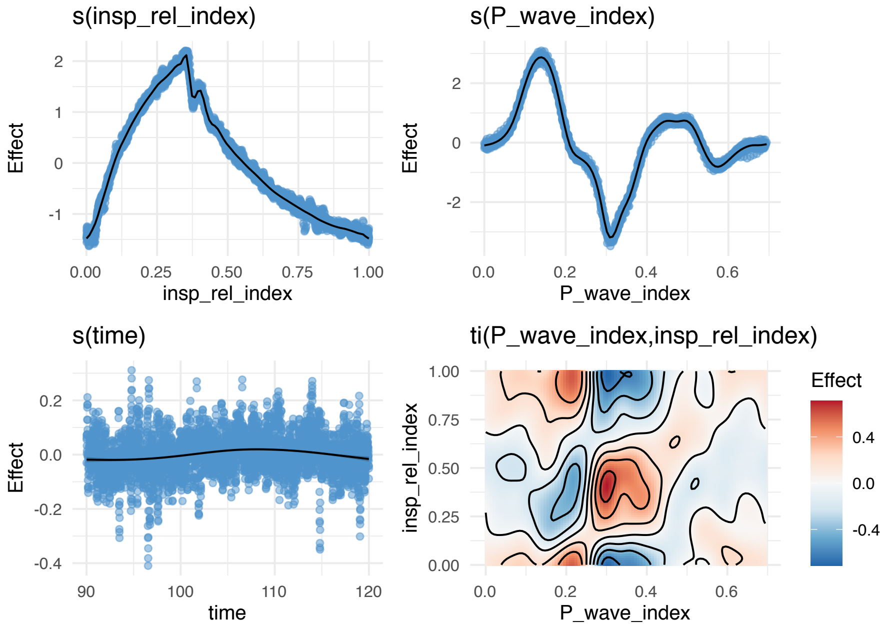


Figure 15: Smooth effects of the CVP GAM with adaptive smoothness.

The disturbance at end-inspiration is now fitted in the respiratory cycle smooth.

### 4.4 Autocorrelated residuals

One assumption of a GAM is that the residuals are independent. This will rarely be true when modelling high resolution waveforms. We can investigate the serial correlation of model residuals using `acf()`.

```
auto_corr_gam_cvp_ad <- acf(residuals(gam_cvp_ad))
```

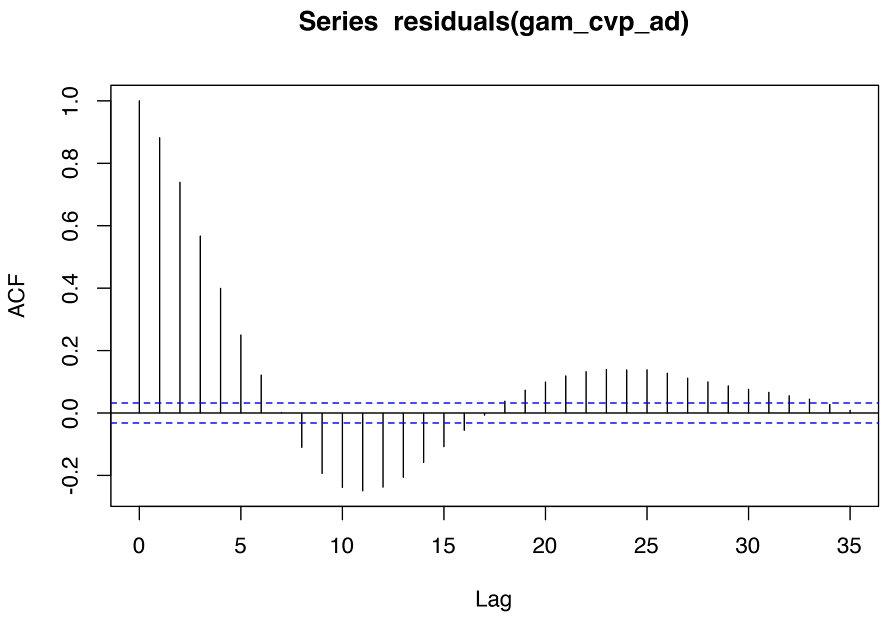


Figure 16: Serial correlation of residuals from the GAM above (gam_cvp_ad). The plot shows the correlation of residuals with the residual 0 to 35 points later. Zero points later correspond to the residual itself, and will always be 1.

`bam()` allows modelling the residual errors as an AR(1) model (each residual ($\epsilon_t$) is some proportion (rho) of the previous residual + random error: $\epsilon_t = rho \times \epsilon_{t-1} + w_t, w_t \sim normal(0, \sigma)$)). `bam()` allows us to specify a fixed AR(1) correlation coefficient with the `rho` parameter, but we have to tune it manually. A good guess is the first-order autocorrelation from a model not accounting for autocorrelation as suggested by van Rij et al, 2019 (https://doi.org/10.1177/2331216519832483).

```
(ar1 <- auto_corr_gam_cvp_ad$acf[2]) # $acf[1] is the unlagged correlation (= 1)
```

```
## [1] 0.8816768
```

The AR(1) model implies that the correlation between residuals drops exponentially with the distance between them. Our CVP waveform has a sample rate of 125 Hz, so a correlation coefficient of 0.88 per sample corresponds to the following relation between correlation and time:

```
time_ms <- 0:500
correlation <- ar1^((time_ms/1000)*125)
```

```r
plot(time_ms, correlation, type = "l",
     main = sprintf("Expected autocorrelation given the AR(1) model
with rho = %0.2f at 125 Hz", ar1))
```

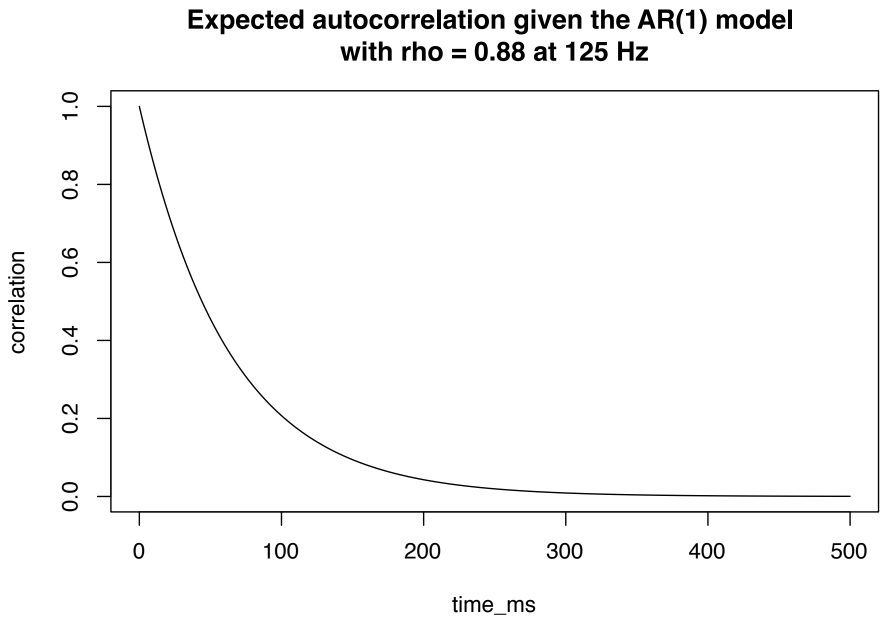


With this model, we expect very little correlation between residuals more than 200 ms apart. We can now fit a GAM that expects autocorrelated residuals.

```r
# The model is identical to gam_cvp_ad except for the rho parameter.
gam_cvp_ad_AR <- bam(
      CVP ~
        s(P_wave_index, bs = "cr", k = 40) +
        s(insp_rel_index, bs = "ad", k = 60, m = 5, xt = list(bs = "cc")) +
        ti(
          P_wave_index,
          insp_rel_index,
          bs = c("cr", "cc"),
          k = c(40, 30)
        ) +
        s(time, bs = "cr"),
        knots = list(insp_rel_index = c(0,1)),
        rho = 0.88, # correlation coefficient for AR(1) model of the residuals
        gamma = 5,
        data = cvp_df_30
    )
```

```r
gratia::draw(gam_cvp_ad_AR, residual = TRUE, rug = FALSE)
```

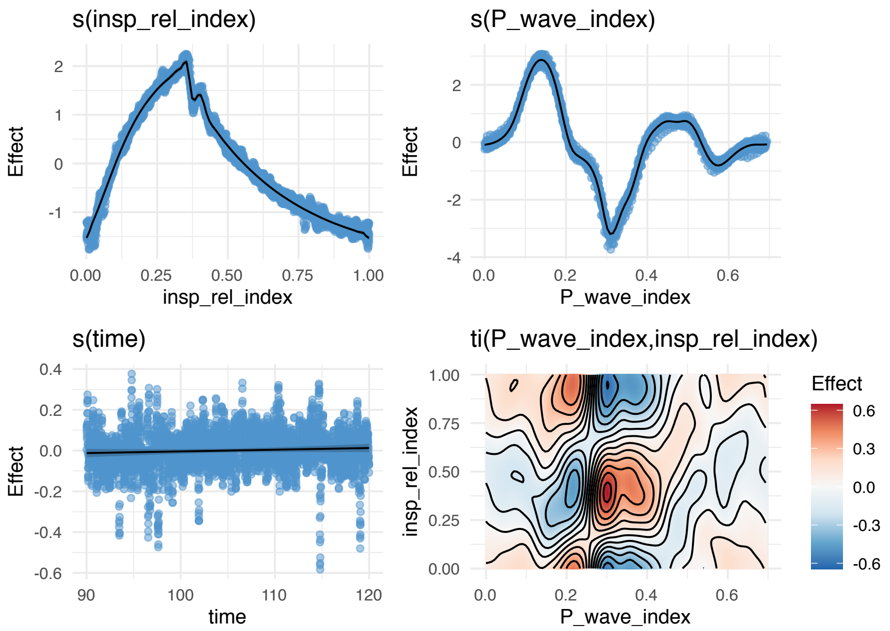


The autocorrelation-corrected residuals are stored in `gam_cvp_ad_AR$std.rsd`. Again, we can use `acf()` to visualise the residual autocorrelation.

```
acf(gam_cvp_ad_AR$std.rsd)
```

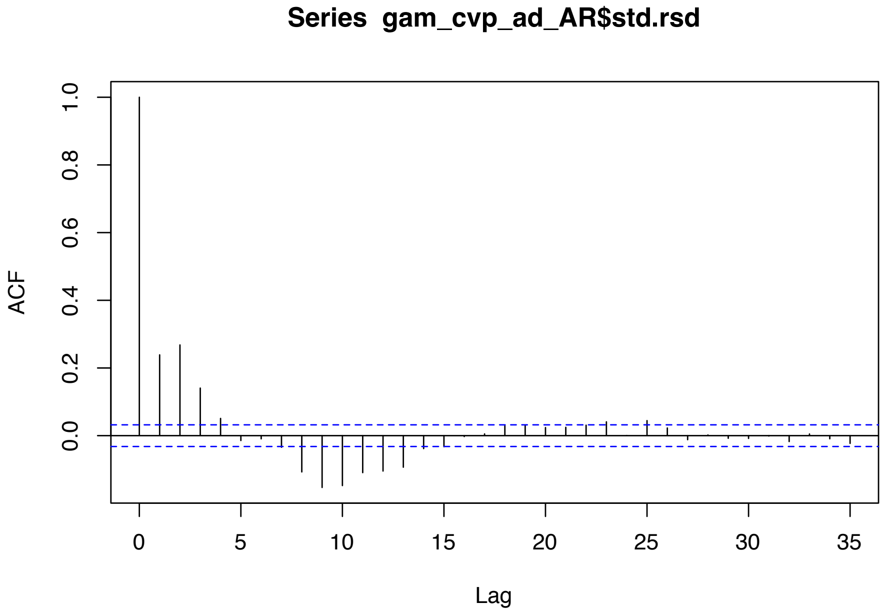


We can see that this has markedly reduced the autocorrelation in the residuals. The `rho` chosen with this approach is not guaranteed to be optimal. It is possible to search for the value of rho minimizing the REML-score, but this is computationally expensive as it requires refitting the model for each rho. For detail about estimating autocorrelation of residuals, see Simpson, 2018 (https://doi.org/10.3389/fevo.2018.00149).

## 4.5 The full model with two sections of data: before and after fluid

To fit the model in the paper's Fig. 6, we first create a data frame that contains both 1-minute sections (before and after fluid).

```
# Section before fluid
cvp_pre <- cvp_df %>%
  filter(between(time, sample_cvp$fluid_start - 60, sample_cvp$fluid_start)) %>%
  mutate(time_s = time - time[1])

# Section after fluid
cvp_post <- cvp_df %>%
  # Select segment starting 60 seconds after fluid administration ends,
  # to get more steady data
  filter(between(time, sample_cvp$fluid_end + 60, sample_cvp$fluid_end + 120)) %>%
  mutate(time_s = time - time[1])

# Combine sections
cvp_pre_post <- bind_rows("pre fluid" = cvp_pre,
                          "post fluid" = cvp_post,
                          .id = "section") %>%
  mutate(section_f = factor(section, levels = c("pre fluid", "post fluid"))) %>%
  group_by(section_f) %>%
  # Create a variable that marks the start of a new section
  # (used to indicate that residuals are not expected to be correlated between the end of one
  # section and the beginning of the next).
  mutate(section_start = row_number() == 1) %>%
  ungroup()
```

With the dataset ready, we can fit the model:

```
gam_cvp_fluid <- bam(
          CVP ~
            # In addition to the intercept, we fit an additional constant for
            # all but the first section (here this is section_f = "post fluid").
            section_f +
            # With the `by` parameter, a separate smooth is estimated for each level of
            # the given factor `section_f`.
            s(P_wave_index, bs = 'cr', k = 40, by = section_f) +
            s(insp_rel_index, bs = "ad", k = 60, m = 5,
              xt = list(bs = "cc"), by = section_f) +
            ti(
              P_wave_index,
              insp_rel_index,
              bs = c('cr', 'cc'),
              # We reduce the complexity of the model by reducing the
              # dimensions of the interaction term.
              k = c(30, 30),
```

```r
            by = section_f
          ) +
          s(time_s, by = section_f),
        knots = list(insp_rel_index = c(0,1)),
        nthreads = 4,
        rho = 0.94,
        # The AR.start parameter marks breaks in the autocorrelation structure.
        AR.start = section_start,
        # In this example we use the gamma parameter to enforce extra smoothing
        # of the model.
        gamma = 5,
        data = cvp_pre_post,
)
```

```r
gratia::draw(gam_cvp_fluid, residuals = TRUE, rug = FALSE)
```

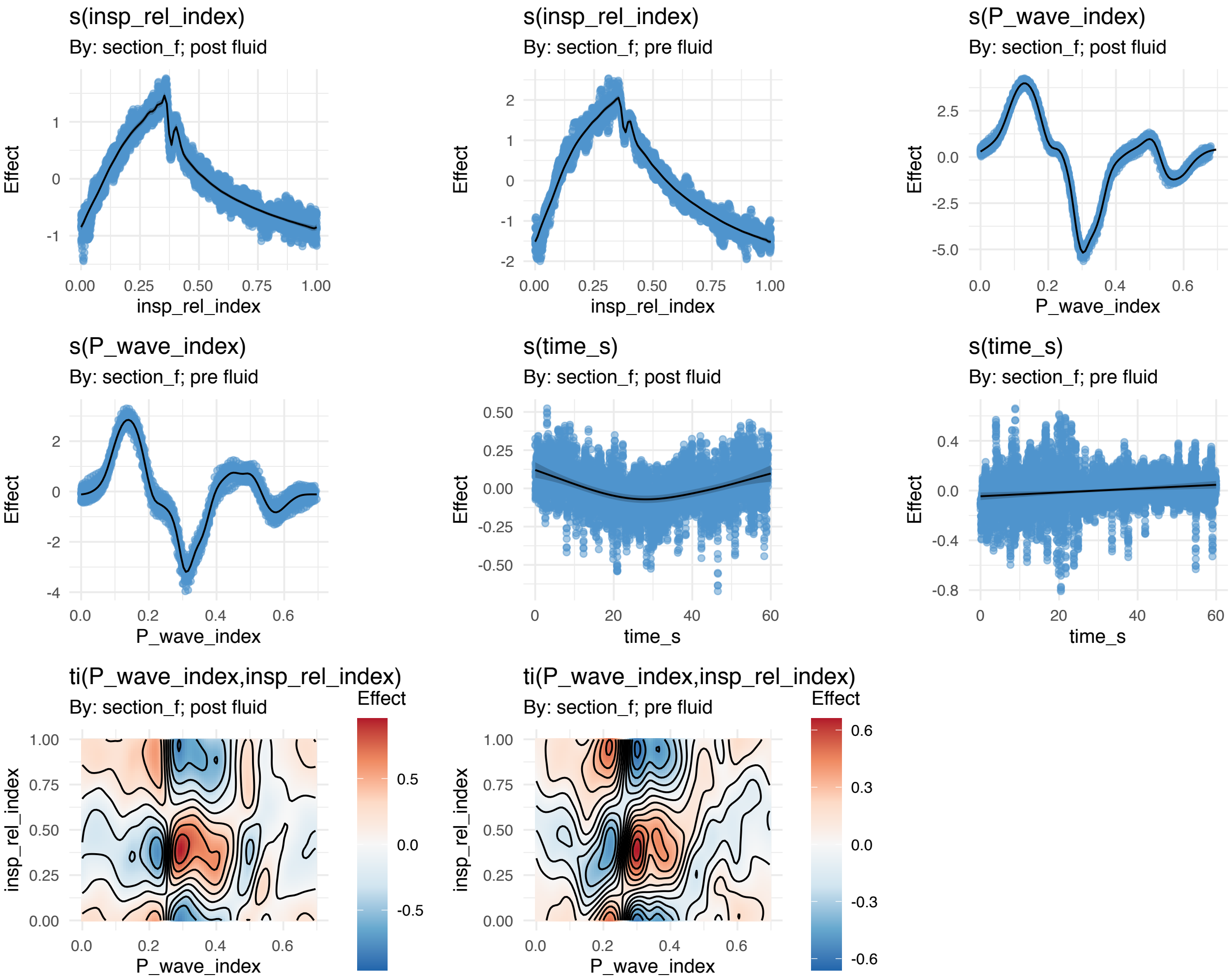


Figure 17: Smooth effects of the GAM of CVP before and after fluid.

In the paper's Fig. 6 the fit is visualised as two contour plots that sum all the effects except

detrending (`s(time_s)`). Here is a similar visualisation:

```
# Create a new dataset containing a grid of
# `P_wave_index` and `insp_rel_index` to generate predictions over.

# Use a grid with a resolution of 100x100.
cvp_contour_data <- expand.grid(
  section_f = factor(c("pre fluid", "post fluid"), levels = c("pre fluid", "post fluid")),
  P_wave_index = seq(0, 0.7, length.out = 100),
  insp_rel_index = seq(0, 1, length.out = 100),
  # We will not use `time` in our prediction, but predict.gam() expects the
  # variable to be present.
  time_s = 9999)



cvp_contour_data$CVP_pred <- predict(gam_cvp_fluid,
                                     newdata = cvp_contour_data,
                                     exclude = c("s(time_s):section_fpost fluid",
                                                 "s(time_s):section_fpre fluid"))

ggplot(cvp_contour_data, aes(x = P_wave_index, y=insp_rel_index, z = CVP_pred)) +
  geom_raster(aes(fill = CVP_pred)) +
  geom_contour(binwidth = 1, color = "black") +
  scale_fill_viridis_c() +
  guides(fill = guide_colorbar(barheight = 15, title = "CVP")) +
  facet_wrap(vars(section_f))
```

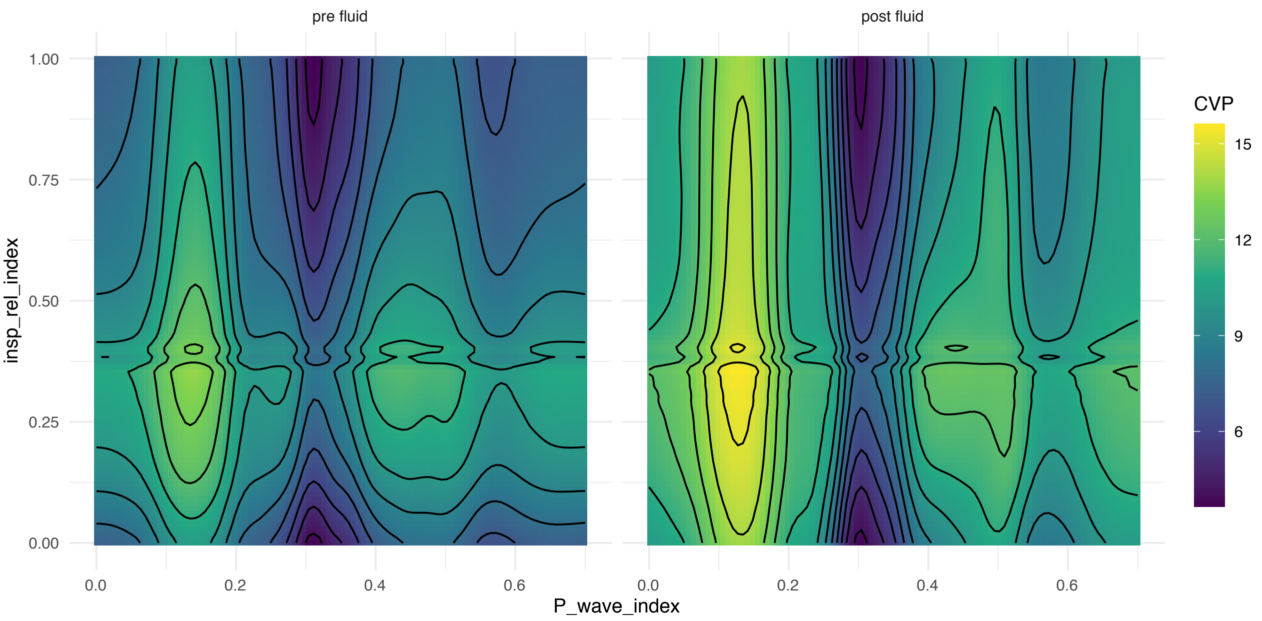


Figure 18: Combined visualization of the GAM of CVP before and after fluid, excluding the detrending smooth.

## 4.6 Other common challenges

There is more noise in the CVP waveform in the 30 seconds before the green area in Figure 10. We will use that section of data to demonstrate a few common challenges.

```
cvp_df_noise <- sample_cvp$cvp %>%
  filter(between(time, sample_cvp$fluid_start-60, sample_cvp$fluid_start-30)) %>%
  # QRS time - PQ interval = P wave time.
  add_time_since_event(sample_cvp$qrs$time - PQ_interval, prefix = "P_wave") %>%
  add_time_since_event(sample_cvp$insp_start$time, prefix = "insp")


cvp_time <- ggplot(cvp_df_noise, aes(time, CVP)) +
  geom_line()

cvp_insp <- ggplot(cvp_df_noise, aes(insp_rel_index, CVP, group = insp_n)) +
  geom_line()

cvp_p <- ggplot(cvp_df_noise, aes(P_wave_index, CVP, group = P_wave_n)) +
  geom_line()

cvp_time / (cvp_insp + cvp_p)
```

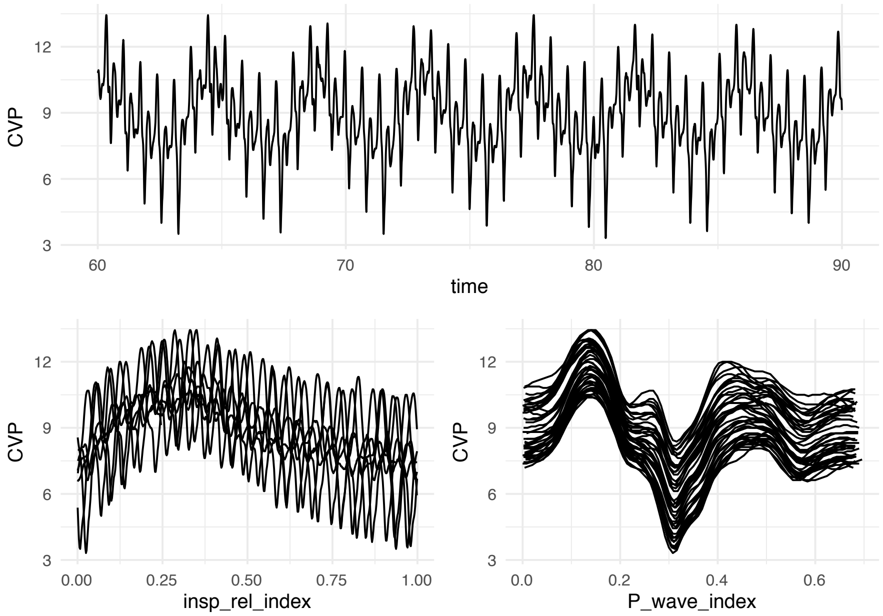


Figure 19: Different visualizations of the noisy data used for the second CVP model.

In the first iteration, we will use fewer knots in the cardiac smooth.

```r
gam_cvp_noise1 <- bam(
      CVP ~
        s(P_wave_index, bs = "cr", k = 15) + # only 15 knots
        s(insp_rel_index, bs = "ad", k = 60, m = 5,
              xt = list(bs = "cc")) +
        ti(
          P_wave_index,
          insp_rel_index,
          bs = c("cr", "cc"),
          k = c(15, 30) # only 15 knots in the cardiac dimension
        ) +
        s(time, bs = "cr"),
        knots = list(insp_rel_index = c(0, 1)),
        data = cvp_df_noise
    )
```

```r
gratia::draw(gam_cvp_noise1, residuals = TRUE, rug = FALSE)
```

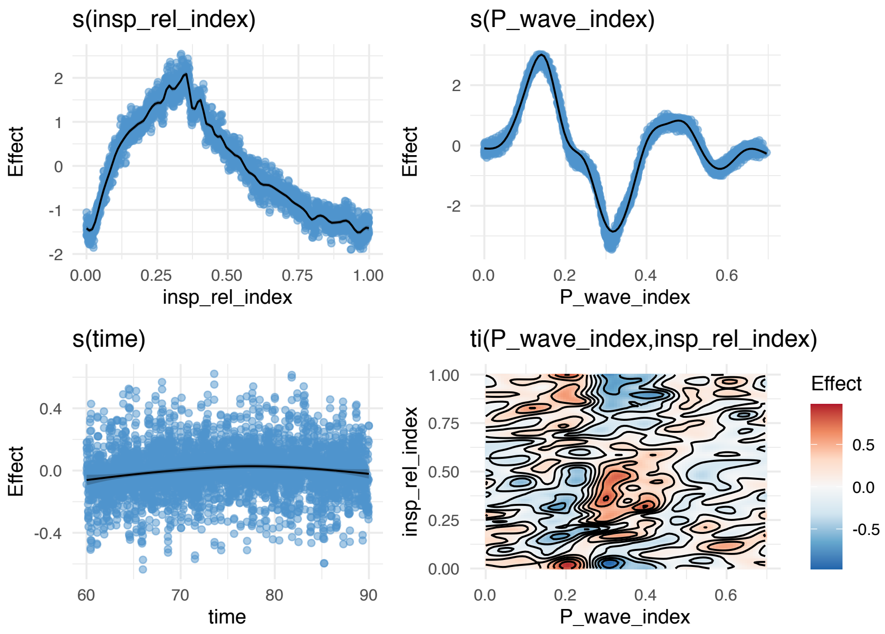


Figure 20: Smooth effects of the noisy CVP GAM.

Here, the interaction smooth clearly overfits the data, and it looks like the cardiac smooth is not flexible enough to match the sharp x' peak (the most negative peak in this sample).

Optimally, the shape of a spline should be limited by the wiggliness penalty (smoothing parameter) and not the number of knots. We can use `k.check` the see if any splines are limited by their number of knots.

```r
k.check(gam_cvp_noise1)
```

```
##                                     k'       edf   k-index p-value
## s(P_wave_index)                     14  13.99274 0.4531906       0
## s(insp_rel_index)                   58  46.29579 1.0983382       1
## ti(P_wave_index,insp_rel_index) 392 318.66971 0.3972915       0
## s(time)                              9   2.58931 0.1592074       0
```

In this table, `k'` is the maximum possible degrees of freedom (d.f.) of the smooth. This is one less than the number knots, as one d.f. is used to constrain the spline to have a sum of zero (see `?mgcv::identifiability`). For cyclic splines it is two less than `k`, as the two limiting knots are effectively a single knot. `edf` is the effective degrees of freedom after the smoothing penalty. A `k-index` $< 1$ indicates there is some residual pattern that is not contained in the smooth. If `k-index` is low and `edf` is close to `k'`, the smooth probably has too few knots. On the other hand, excessive knots is not a problem, (except for the higher computational cost).

We increase `k` to 40 for the cardiac smooth and the cardiac dimension in the interaction. To reduce overfitting from the interaction term, we increase `gamma` to make the model smoother.

```r
gam_cvp_noise2 <- bam(
      CVP ~
        s(P_wave_index, bs = "cr", k = 40) + # k has been increased
        s(insp_rel_index, bs = "ad", k = 60, m = 5,
              xt = list(bs = "cc")) +
        ti(
          P_wave_index,
          insp_rel_index,
          bs = c("cr", "cc"),
          k = c(40, 30) # k has been increased for the cardiac dimension
        ) +
        s(time, bs = "cr"),
        knots = list(insp_rel_index = c(0,1)),
        data = cvp_df_noise,
      gamma = 5
    )
```

```r
gratia::draw(gam_cvp_noise2, residuals = TRUE, rug = FALSE)
```

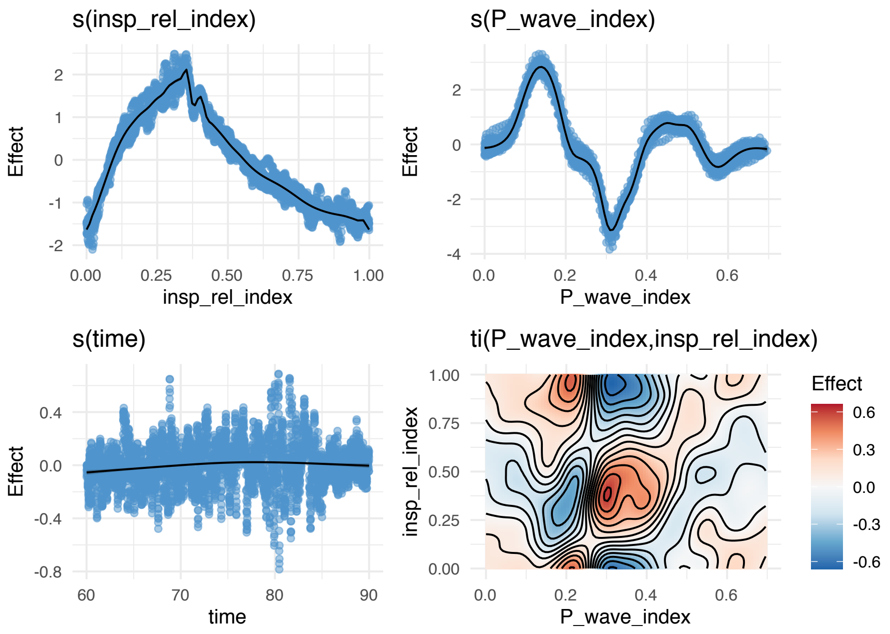


Figure 21: Smooth effects of a revised version the noisy CVP GAM. This time with more knots in the cardiac smooth (s(P__wave__index)) and fixed smoothing parameters of the interaction smooth.

```
cvp_df_noise_pred2 <- mutate(cvp_df_noise,
                             pred = predict(gam_cvp_noise2),
                             resid = resid(gam_cvp_noise2))

ggplot(cvp_df_noise_pred2, aes(time, CVP)) +
  geom_line() +
  geom_line(aes(y = pred), color = "red")+
  geom_line(aes(y = resid), color = "orange")
```

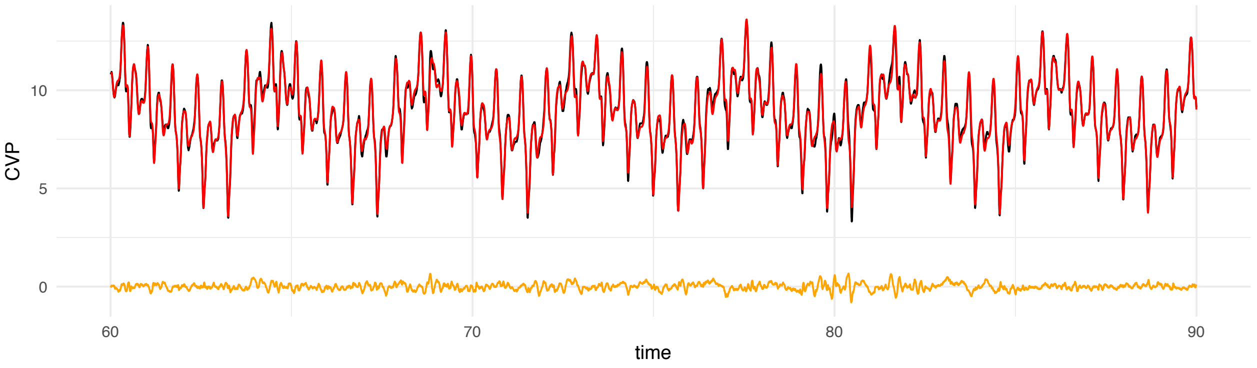


# 5 Session info

*This section shows information about the system used to generate this pdf file*

Rendered: 2022-03-22 13:43:24

```r
sessionInfo()
```

```
## R version 4.1.0 (2021-05-18)
## Platform: x86_64-pc-linux-gnu (64-bit)
## Running under: Pop!_OS 21.10
##
## Matrix products: default
## BLAS/LAPACK: /usr/lib/x86_64-linux-gnu/libmkl_rt.so
##
## locale:
##  [1] LC_CTYPE=en_US.UTF-8       LC_NUMERIC=C
##  [3] LC_TIME=en_DK.UTF-8        LC_COLLATE=en_US.UTF-8
##  [5] LC_MONETARY=en_DK.UTF-8    LC_MESSAGES=en_US.UTF-8
##  [7] LC_PAPER=en_DK.UTF-8       LC_NAME=C
##  [9] LC_ADDRESS=C               LC_TELEPHONE=C
## [11] LC_MEASUREMENT=en_DK.UTF-8 LC_IDENTIFICATION=C
##
## attached base packages:
## [1] stats     graphics  grDevices utils     datasets  methods   base
##
## other attached packages:
## [1] patchwork_1.1.1 ggplot2_3.3.5   dplyr_1.0.8     gratia_0.7.0-5
## [5] mgcv_1.8-36     nlme_3.1-152
##
## loaded via a namespace (and not attached):
##  [1] tidyselect_1.1.2   xfun_0.30          purrr_0.3.4        splines_4.1.0
##  [5] lattice_0.20-44    colorspace_2.0-3   vctrs_0.3.8        generics_0.1.2
##  [9] viridisLite_0.4.0  htmltools_0.5.2    yaml_2.3.5         utf8_1.2.2
## [13] rlang_1.0.2        isoband_0.2.5      pillar_1.7.0       glue_1.6.2
## [17] withr_2.5.0        DBI_1.1.1          RColorBrewer_1.1-2 lifecycle_1.0.1
## [21] stringr_1.4.0      munsell_0.5.0      gtable_0.3.0       mvnfast_0.2.7
## [25] evaluate_0.15      labeling_0.4.2     knitr_1.37         fastmap_1.1.0
## [29] fansi_1.0.2        Rcpp_1.0.8         scales_1.1.1       farver_2.1.0
## [33] digest_0.6.29      stringi_1.7.6      bookdown_0.22      RcppRoll_0.3.0
## [37] grid_4.1.0         cli_3.2.0          tools_4.1.0        magrittr_2.0.2
## [41] tibble_3.1.6       crayon_1.5.0       tidyr_1.2.0        pkgconfig_2.0.3
## [45] ellipsis_0.3.2     Matrix_1.3-4       assertthat_0.2.1   rmarkdown_2.12.2
## [49] rstudioapi_0.13    R6_2.5.1           compiler_4.1.0
```
